# Sodium pump subunit NKAα1 protects against diabetic endothelial dysfunction by inhibiting ferroptosis through the autophagy‐lysosome degradation of ACSL4

**DOI:** 10.1002/ctm2.70221

**Published:** 2025-02-04

**Authors:** Xue‐Xue Zhu, Jia‐Bao Su, Fang‐Ming Wang, Xiao‐Ying Chai, Guo Chen, An‐Jing Xu, Xin‐Yu Meng, Hong‐Bo Qiu, Qing‐Yi Sun, Yao Wang, Zhuo‐Lin Lv, Yuan Zhang, Yao Liu, Zhi‐Jun Han, Na Li, Hai‐Jian Sun, Qing‐Bo Lu

**Affiliations:** ^1^ Department of Basic Medicine Department of Endocrinology Affiliated Hospital of Jiangnan University Jiangnan University Wuxi China; ^2^ Department of Basic Medicine Wuxi School of Medicine Jiangnan University Wuxi Jiangsu China; ^3^ Department of Rheumatology and Immunology Affiliated Hospital of Jiangnan University Jiangnan University Wuxi China; ^4^ Department of Cardiac Ultrasound The Fourth Affiliated Hospital of Nanjing Medical University Nanjing China; ^5^ Department of Clinical Research Center Jiangnan University Medical Center (Wuxi No.2 People's Hospital) Wuxi School of Medicine Jiangnan University Wuxi China; ^6^ Research Institute for Reproductive Health and Genetic Diseases Wuxi Maternity and Child Health Care Hospital Wuxi China; ^7^ State Key Laboratory of Natural Medicines China Pharmaceutical University Nanjing China

**Keywords:** diabetes, endothelial dysfunction, ferroptosis autophagy, lysosome, oxidative stress

## Abstract

The sodium pump Na+/K+‐ATPase (NKA), an enzyme ubiquitously expressed in various tissues and cells, is a critical player in maintaining cellular ion homeostasis. Dysregulation of α1 subunit of NKA (NKAα1) has been associated with cardiovascular and metabolic disorders, yet the exact role of NKAα1 in diabetes‐induced endothelial malfunction remains incompletely understood. The NKAα1 expression and NKA activity were examined in high‐glucose (HG)‐exposed endothelial cells (ECs) and mouse aortae, as well as in high‐fat‐diet (HFD)‐fed mice. Acetylcholine (Ach) was utilised to assess endothelium‐dependent relaxation (EDR) in isolated mouse aortae. We found that both NKAα1 protein and mRNA levels were significantly downregulated in the aortae of HFD‐fed mice, and HG‐incubated mouse aortae and ECs. Gain‐ and loss‐of‐function experiments revealed that NKAα1 preserves EDR by mitigating oxidative/nitrative stresses in ECs. Overexpression of NKAα1 facilitated EC viability, migration, and angiogenesis by inhibiting the overproduction of superoxide and peroxynitrite. Mechanistically, dysfunctional NKAα1 impaired autophagy process, and prevented the transfer of acyl‐CoA synthetase long‐chain family member 4 (ACSL4) to the lysosome for degradation, thereby resulting in lipid peroxidation and ferroptosis in ECs. Induction of ferroptosis and inhibition of the autophagy‐lysosome pathway blocked the protective effects of NKAα1 on EDR. Eventually, we identified Hamaudol as a potent activator of NKAα1 by restraining the phosphorylation and endocytosis of NKAα1, restoring EDR in obese diabetic mice. Overall, NKAα1 facilitates the autophagic degradation of ACSL4 via the lysosomal pathway, preventing ferroptosis and oxidative/nitrative stress in ECs. NKAα1 may serve as an attractive candidate for the management of vascular disorders associated with diabetes.

**Key points:**

- NKAα1 downregulation impairs endothelial function in diabetes by promoting oxidative/nitrative stress and ferroptosis.
- NKAα1 supports lysosomal degradation of ACSL4 via autophagy, preventing lipid peroxidation and ferroptosis.
- Hamaudol, an activator of NKAα1, restores endothelial relaxation in diabetic mice by inhibiting NKAα1 phosphorylation and endocytosis.

## Graphical abstract

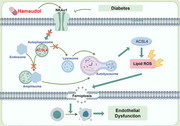

## 1 INTRODUCTION

Endothelial cells (ECs), located in the endothelium of blood vessels, govern vascular tone by secreting nitric oxide (NO), and prostacyclin (PGI2), with NO being the primary vasodilator.^1^, ^2^ In contrast, ECs also release superoxide anion, endothelin 1, and constrictive prostaglandins that lead to vascular contraction.^3^ Disruption of the balance between vasodilatory and vasoconstrictive factors leads to increased oxidative stress, inflammatory response, and reduced NO bioavailability in ECs, ultimately causing endothelial dysfunction.^4^ Endothelial dysfunction is a pathological characteristic in a host of human diseases, resulting in functional disruption of multiple organs.^4^ Restoring EC function has thus been proposed as a promising strategy for treating obesity, diabetes, hypertension, and atherosclerosis.^5^ Diabetes is becoming a serious global public issue with a rising prevalence globally.^6^ Patients with diabetes are at a higher risk of cardiovascular accidents.^7^, ^8^ Although the mechanisms behind this increased risk are unclear, endothelial dysfunction is a central player in diabetic complications.^9^ As such, improvement of endothelial dysfunction is of clinical relevance in diabetes.

Na+/K+‐ATPase (NKA), a multifunctional ion transporter, maintains sodium and potassium ion balance at the cellular membrane.^10^ As a widely expressed protein, NKA plays essential roles in sodium/potassium homeostasis and signal transduction in the cardiac, neurological, hepatic, and renal systems.^11^ This enzyme comprises α, β, and γ subunits.^11^ To date, four isoforms of NKA subunits have been identified. The NKAα1 isoform, the most prevalent, is found in nearly all cell types.^12^ The NKAα2 isoform is mainly expressed in skeletal muscle and adipose tissue.^12^ The NKAα3 isoform is present mainly in neurons and cardiac cells, while the NKAα4 isoform is exclusively expressed in testes.^12^ Studies have shown that genetic inhibition of NKAα1 leads to metabolic disorders in the liver,^13^ doxorubicin‐induced cardiotoxicity,^14^ stress‐induced anxiety,^15^ Parkinson disease,^16^ and brain ischemic injury.^17^ In the vascular system, inhibition of NKA with ouabain contributes to the increased vascular tone and hypertension in rats.^18^, ^19^ Activation of NKA is required for NO‐dependent acetylcholine relaxation in porcine internal mammary artery^20^ and rat aorta.^21^ Activation of NKA is documented to prevent aortic endothelial dysfunction in lead‐treated rats.^22^ Collectively, it is likely that NKA is critically involved in regulating vascular tension. Recently, it has been established that targeting NKAα1 prevents doxorubicin‐induced cardiotoxicity or Parkinson's disease by suppressing the process of ferroptosis.^14^, ^16^ We hypothesise that NKAα1 may maintain endothelial function and prevents ferroptosis in ECs since ferroptosis is a crucial contributor to endothelial dysfunction in diabetes.^23^, ^24^ Also, we hypothesised that activating NKAα1 may restore endothelial function, reduce oxidative/nitrative stress, and serve as a therapeutic approach for diabetes‐related vascular complications. Thereafter, the current study aims to explore whether NKAα isoforms were changed in diabetic endothelial dysfunction, and to investigate whether restored impaired NKAα isoforms protected against diabetes‐induced endothelial dysfunction, and to shed light on the underlying mechanisms involved, especially ferroptosis.

## 2 MATERIALS AND METHODS

### 2.1 Reagents

NKAα1 antibody (sc‐514614) and NKAα3 antibody (sc‐376967), NKAα1 siRNA (sc‐36010) and control siRNA (sc‐36869) were obtained from Santa Cruz. Primary antibodies against β‐actin (81115‐1‐RR), NKAα2 (16836‐1‐AP), gp91^phox^ (82872‐1‐RR), ACSL4 (22401‐1‐AP), Cathepsin B (12216‐1‐AP), and LAMP1 (67300‐1‐Ig) were sourced from Proteintech Group. Primary antibodies against GPX4 (ab125066), p62 (ab109012), pan‐cadherin (ab51034), LGALS3 (ab2785), LC3B (ab192890), and P‐NKAα1 (ab194532) were procured from Abcam. EdU cell proliferation imaging kit containing Alexa Fluor ^™^ 488 dye (C10337), and TUNEL detection kit, Alexa Fluor ™ 594 dye (C10618) were obtained from Thermo Fisher Scientific. NKAα1 human clone plasmid (RC201009) and scrambled plasmid (GE100003) were bought from ORIGENE. 4′,6‐Diamidine‐2′‐phenylindole dihydrochloride (DAPI) (C1002) and dihydroethidium (DHE) (S0063) were gained from Beyotime Biotechnology. 3‐MA (5142‐23‐4), FerroOrange (SCT210), sodium nitroprusside (BP453), acetylcholine (PHR1546), chloroquine (C6628), phenylephrine (Phe, P1240000), D‐glucose (PHR1000), and cycloheximide (CHX, 66‐81‐9) were purchased from Sigma. Ferrostatin‐1 (Fer‐1, HY‐100579), Z‐VAD‐fmk (HY‐16658B), Z‐YVAD‐fmk (HY‐P1009), necrostatin‐1 (Nec‐1, HY‐15760), and Tetrathiomolybdate (HY‐128530) were acquired from MedChemExpress. The kits for triglyceride (TG, A110‐1‐1), total cholesterol (TC, A111‐1‐1), and insulin (H203‐1‐2) were purchased from Jiancheng Institute (Nanjing, China). The selected concentrations of chloroquine (100 µM), Nec‐1 (10 µM), Z‐VAD‐fmk (25 µM), MG‐132 (10 µM), Fer‐1 (5 µM), Z‐YVAD‐fmk (10 µM), or Tetrathiomolybdate (1 µM) were based on the previous reports.^13^, ^25^

### 2.2 Animals

All experiments in mice were reviewed and approved by Animal Care and Use Committee of the China Pharmaceutical University (202101017). The animals were kept in an environment with a stable temperature. NKAα1 heterozygous (KAα1*^+/−^*) mice were provided by University of Cincinnati.^26^ Wile‐type (WT) C57BL/6J mice and KAα1*^±^* mice, aged of 8 weeks, were subjected to a high‐fat diet (HFD, 60% kcal as fat, Research Diets) for continuous 12 weeks as described previously.^13^, ^27^, ^28^ Age‐matched control mice were fed a normal chow diet for the same duration. Mice received intravenous injections of AAV5‐TIE1‐NKAα1 overexpression plasmid (10^11^ viral genome particles for each mouse) or negative control AAV5 vectors after 6 weeks of HFD feeding. After six weeks, the aortae were harvested for functional analyses. Euthanasia was performed by anaesthetising the animals with 5% isoflurane. For ex vivo experiments, the aortic rings (2 mm) were isolated from 8‐week‐old WT and NKAα1*^+/−^* mice and cultured in Dulbecco's modified Eagle medium (DMEM, Gibco, 11960044) coupled with 10% fetal bovine serum (Gibco, 10099–141C), penicillin (100 IU/mL) and streptomycin (100 µg/mL). High‐glucose (HG) conditions were constructed by the presence of glucose (25 mM), whereas mannitol (25 mM) was applied as the normal glucose (NG) osmotic control. After 48 h, the aortic rings were mounted onto a myograph for the assessment of vascular tone. Six weeks after tail vein injection of AAV5‐TIE1‐NKAα1 overexpression plasmids (10^11^ viral genome particles per mouse) in normal mice, the mouse aortae were then incubated with NG or HG for additional 48 h to evaluate the effects of endothelial cell‐specific overexpression of NKAα1 on vascular functions in diabetes.

### 2.3 Vascular function measurement

After sacrifice, the isolated mouse thoracic aortae were placed in an oxygenated, ice‐cold physiological saline solution (PSS). The mouse aortae were then cut into 2‐mm rings and mounted on stainless steel wires (Model 620 M, Aarhus, Denmark) containing 5 mL of aerated PSS at 37°C. Arterial segments were stretched to a tension of 3 mN and equilibrated for 60 min before the experiments. Following equilibration, the mouse vascular rings were precontracted with 1 µM phenylephrine (Phe) and subsequently rinsed. Endothelium‐dependent relaxation (EDR) was measured by adding various concentrations of acetylcholine (Ach, 10^−9^ to 10^−5^ M) to Phe‐precontracted rings. Endothelium‐independent relaxation was evaluated by applying different doses of sodium nitroprusside (SNP, 10^−9^ to 10^−5^ M) to rings where the endothelium was removed by gentle rubbing.

### 2.4 Western blotting

Proteins from cells and mouse aortae were isolated using prechilled lysis buffer (P0013, Beyotime Biotechnology, Shanghai, China). The protein concentrations in each sample were measured with a BCA kit (P0012S, Beyotime Biotechnology, Shanghai, China) after centrifugation at 12 000 rpm for 15 min at 4°C. Equal protein amounts were separated by SDS‐PAGE, and transferred to PVDF membranes (03010040001, Millipore), and blocked with 5% skim milk for 1 h. Primary antibodies were added into the PVDF membranes overnight at 4°C. After incubation of HRP‐conjugated secondary antibodies for 1 h at room temperature, the blots were obtained using enhanced chemiluminescence (BL523B, Biosharp).

### 2.5 Quantitative real‐time (RT) PCR

Total RNA in each sample was isolated with Trizol reagents (Vazyme, R711‐01) following the manufacturer's protocols. Subsequently, 1 µg of RNA per sample was reversely transcribed using Hifair® III first Strand cDNA Synthesis SuperMix (Yeasen, 11120ES60). Real‐time PCR was carried out on an Applied Biosystems QuantStudio 5 PCR system (ThermoFisher, USA), and mRNA levels were normalised to the housekeeping genes with the aid of the 2^−∆∆Ct^ method. Detailed information for the used primer sequences was provided in Tables S1 and S2.

### 2.6 Measurement of NKA activity

The activity of NKA was measured as we previously depicted.^29^ Samples were randomly divided into two 50 µL homogenate aliquots. One sample was incubated with the reaction buffer without ouabain, and the other sample with buffer containing 2 mM ouabain. The reactions were initiated with 1 mM ATP, stopped with 10 µL of 100% trichloroacetic acid for 60 min. Free phosphates were measured at 650 nm using a spectrophotometric assay kit.

### 2.7 Cell culture

Primary endothelial cells (ECs) from mouse aortae were conducted as previously documented.^2^, ^28^, ^30^, ^31^ Briefly, mice were anaesthetised with 5% isoflurane. Heparin (100 U/mL) was injected into the left ventricle. Mouse aortae were incubated in DMEM with .8 mg/mL collagenase type II (Sigma) for 8 min at 37°C. Detached ECs were collected by centrifugation (1000 rpm, 5 min), re‐suspended in DMEM with 20% fetal bovine serum, and cultured in endothelial growth medium (Pricella, CM‐M075) until confluency. Primary vascular smooth muscle cells and vascular fibroblasts were cultured as previously described.^32^, ^33^, ^34^ Human umbilical vein endothelial cells (HUVECs, CL‐0675, Pricella, Wuhan, China) were cultured in RPMI 1640 medium (Gibco, 11875093) supplemented with fetal bovine serum (10%), 100 U/mL penicillin, and 100 µg/mL streptomycin at 37°C and 5% CO_2_. To simulate diabetic conditions, HUVECs were treated for 24 h with high‐glucose/high‐fat (HG/HF) medium containing 25 mM glucose (Sigma) and 500 µM palmitate (Sigma) following the established protocols.^35^, ^36^

### 2.8 Cell counting kit‐8 (CCK‐8) assay

Cell viability was determined using the commercial CCK‐8 kits (Beyotime, C0038) following the manufacturer's protocols. Treated cells were seeded in 96‐well plates, incubated with the stock CCK‐8 solution (10 µL) for 2 h at 37°C, and the absorbance was assessed at 450 nm using a microplate reader.

### 2.9 DHE staining

The intracellular ROS levels were quantified using the DHE probe. In brief, treated cells were challenged by DHE probe (10 µM) at 37°C for 30 min under the dark environment. After that, the images were captured by a fluorescence microscope (Zeiss, Germany).

### 2.10 Determination of glutathione (GSH) contents

Intracellular GSH levels were assessed with the aid of a commercial kit (Oxis International Inc., BIOXYTECH GSH‐400) In short, samples were lysed in 5% metaphosphoric acid, centrifuged at 12 000 × *g* for 15 min, and the samples were then collected. GSH levels were measured at 400 nm using a microplate reader.

### 2.11 Determination of malondialdehyde (MDA)

Lipid peroxidation was quantified by using a commercial MDA assay kit (A03‐1‐2, Nanjing Jiancheng Bioengineering Institute), where MDA reacts with TBA at 95°C under acidic conditions to form a pink adduct. Absorbance was measured at 450 nm, and MDA concentration calculated per the manufacturer's instructions.

### 2.12 Determination of Fe^2+^ levels

Intracellular Fe^2+^ levels were measured using the FerroOrange probe (DOJINDO, F374). Briefly, treated cells were rinsed twice with sterilised PBS before incubation with the Hoechst (H342, DOJINDO) for 10 min. After that, cells were stained with FerroOrange solution (1:1000 dilution) in the dark at 37°C for 30 min with 5% CO_2_. The cells without washing were analysed using a fluorescence microscope (Zeiss, Germany). In addition, Fe^2+^ levels were also measured using commercial kits from Abcam (ab83366).^37^, ^38^ A total of 100 µL of iron assay buffer was added and homogenised cells or tissues on ice. The supernatant in the mixture was obtained after centrifugation at 16 000 × *g* for 10 min. A total of 50 µL of each sample were added to 96‐well plates and adjusted to 100 µL using iron assay buffer. For divalent iron assays, 5 µL of buffer was then added, and for total iron assays, 5 µL of iron reducer. After a 30‐min incubation at 37°C, 100 µL of Iron Probe was added, followed by another 60‐min incubation. The absorbance was measured at 593 nm using a microplate reader, and iron concentration was calculated from a standard curve.

### 2.13 Mass spectrometry (MS) analysis of lipidomics

Lipids from approximately 5 × 10⁶ cells were extracted following the Bligh and Dyer method.^39^ After drying, the organic phase was re‐dissolved in 50 µL of mobile phase A [trichloromethane/isopropanol 2:1 (v/v)] and vortexed. The phospholipids were then combined with mobile phase B (acetonitrile/isopropanol/water 7:9:4, v/v/v) containing 10 mM ammonium acetate. MS and MS/MS analyses were conducted using a Q‐Exactive quadrupole‐orbitrap mass spectrometer (Thermo Fisher, Waltham, MA, USA).

### 2.14 Detection of superoxide anions

The production of superoxide anions, indicative of oxidative stress, was quantified in aortic segments or ECs using lucigenin‐enhanced chemiluminescence assay as we previously outlined.^40^ The contents of superoxide anions were expressed in relative light units (RLU) per milligram of protein per second (RLU/s/mg).

### 2.15 Measurement of total NO_x_ and nitrotyrosine

Basal NO_x_ (NO, NO_2_⁻, and NO_3_⁻) production in cells or mouse aortae was measured by collecting supernatants and using a NO assay kit (Beyotime Biotechnology, S0023) following the manufacturer's protocols. Protein concentration was calculated with a BCA protein assay kit. Nitrotyrosine levels, indicative of protein nitration, were quantified using a Nitrotyrosine Assay Kit (Millipore, 17–376RF) and the results were expressed as pM per mg protein (pM/mg protein).

### 2.16 Measurement of caspase 3 activity

The caspase‐3 activity assay kit (#5723, Cell Signaling Technology) was utilised to measure the activity of caspase‐3 in compliance with the manufacturer's indications.^41^ After washing, fluorescence was detected using an ELISA plate reader with excitation at 380 nm and emission at 450 nm. The activity of caspase‐3 was calculated based on the fluorometric signal (excitation/emission = 380/450 nm) and expressed as fold‐change compared to the normal group.

### 2.17 5‐Ethynyl‐2′‐deoxyuridine (EdU) incorporation analysis

HUVEC proliferation was also examined using the EdU cell proliferation kit containing Alexa Fluor ^™^ 488 dye following the manufacturer's protocols. After treatment, HUVECs were incubated with 50 mM EdU for 2 h, then fixed, permeabilised, and stained with Apollo for 30 min. Nuclear DNA was counterstained with Hoechst 33342, and fluorescent images were captured using a microscope.

### 2.18 Terminal deoxynucleotidyl transferase‐mediated dUTP nick end‐labelling (TUNEL) analysis

The apoptosis in HUVECs was assessed by using the TUNEL detection kit in conjunction with Alexa Fluor ™ 594 dye. The treated cells were fixed with 4% paraformaldehyde, permeabilised using .1% Triton X‐100, and washed with PBS. After a 1‐h incubation at 37°C with TF3‐dUTP, nuclei were stained with Hoechst, and TUNEL‐positive cells were captured via a fluorescence microscopy.

### 2.19 Transwell migration assay

HUVEC migration was assessed using a transwell chamber (Corning, CLS3422). Cells (3 × 10^4^/well) were seeded in the upper chamber with serum‐free DMEM, while the lower chamber containing DMEM with 10% fetal bovine serum. After 24 h, nonmigrated cells were removed with cotton swabs, and migrated cells were stained with crystal violet (.1%). The number of migrated cells was captured and counted under a phase‐contrast microscope (80i, Nikon, Japan).

### 2.20 Tube formation assay

Ice‐cold Matrigel (BD Biocoat, 356234) was added to the required wells of a 96‐well plate at 37°C for 30 min. HUVECs (1 × 10^4^ cells/well) were then seeded. The tube formation was captured in each well using a microscope (Olympus, Japan). ImageJ software was utilised for experimental analysis of tube formation, such as the branching points formed by endothelial cells, average tube length, total tube length, and average area of the annular structure.

### 2.21 Immunofluorescent staining in HUVECs

Immunofluorescent staining was used to localise nitrotyrosine in HUVECs. The fixed cells were incubated with an anti‐3‐nitrotyrosine antibody (Abcam, ab314438) overnight. After washing, cells were probed with Alexa Fluor™ 594‐conjugated Goat anti‐Rabbit IgG for 1 h. For double staining, primary antibodies against ACSL4, LGALS3, or LAMP1 were applied overnight, followed by the indicated secondary antibodies. Nuclei were re‐stained with DAPI (5 µM) for 5 min, and images were captured with an Olympus microscope (Japan).

### 2.22 Measurement of eNOS activity

The eNOS activity in ECs and mouse aortae was measured by calculating the transformation of L‐arginine to NO using a commercial kit (S0025, Beyotime Biotechnology, Shanghai, China), as previously described.^42^, ^43^

### 2.23 Enzyme‐linked immunosorbent assay (ELISA)

The levels of TNF‐α (EK0525, EK0527), IL‐1β (EK0392, EK0394), VCAM‐1 (EK0537, EK0538), and MCP‐1 (EK0441, EK0568) in cells and mouse serum were measured using commercial ELISA kits (BOSTER, Wuhan, China).^44^, ^45^ Samples, diluted standards, or blanks were added to 96‐well plates coated with specific antibodies, and HRP‐conjugated secondary antibodies were incubated at 37°C for 30 min. The reactions were stopped with a stop solution, and the absorbance was assessed at 450 nm via a microplate reader (SYNERGY/H4, BioTek, Vermont, USA).

### 2.24 Cell transfection

Cells were seeded to achieve an optimal density of 80–90% confluence. Cell transfection was performed using Lipofectamine 3000 reagent (Thermo Fisher Scientific, L3000075) with control siRNA, NKAα1 siRNA, empty vectors, or NKAα1 overexpression plasmid. After transfection, the cells were incubated for 6 h before replacing the medium with complete medium. After transfection for 24 h, the cells were then subjected to the indicated treatments. ECs with NKAα1 overexpression and suppression were confirmed by Western blotting.

### 2.25 Detection of autophagy

Autophagy monitoring was performed as we previously described.^13^ In short, HUVECs were transfected with a plasmid expressing EGFP‐LC3 using Lipofectamine 3000 to monitor autophagy flux. The LC3 dots were photographed using a laser confocal microscope (Fluoview FV1000, Olympus).

### 2.26 Molecular docking

The protein structure of NKAα1 (PDB: 7E1Z) was optimised using MOE 2019 software. The molecular structure of Hamaudol was retrieved from the PubChem database and imported into Chem3D. For binding affinity assessment, the Hamaudol ligands and protein files were uploaded separately to SwissDock for molecular docking. After optimising the configuration using MM2 force field, semi flexible docking was performed using MOE 2019 software, and the docking results were evaluated using the London dG scoring function. Two‐dimensional (2D) interaction predictions between NKAα1 and Hamaudol were performed using MOE 2019 software.

### 2.27 Extraction of cell membrane and cytoplasmic proteins

Membrane and cytoplasmic proteins were extracted using a commercial protein extraction kit (P0033, Beyotime Biotechnology). Samples were incubated with 1 mL of membrane protein extraction reagent A on ice for 15 min, then homogenised in an ice‐cold glass homogeniser. After 50 strokes, the homogenates were centrifuged at 700 × *g* for 10 min at 4°C, and the supernatants were obtained without disturbing the pellet. The samples were centrifuged at 14 000 × *g* at 4°C for 30 min to pellet the cell membrane fragments. The supernatant contains cytoplasmic proteins. A total of 30–50 µL of supernatant was left to avoid contamination with the pellet. The supernatants containing membrane proteins were centrifuged at 14 000 × *g* for 10 s. After vortexing and incubation 1–2 times to extract membrane proteins thoroughly, the supernatants were centrifuged at 14 000 × *g* for 5 min, and the supernatants containing membrane proteins were then obtained.

### 2.28 Lysotracker DND‐99 staining of lysosomes

HUVECs were cultured on six‐well chambered coverslips, treated accordingly, and incubated with 1 µM Lysotracker DND‐99 (Invitrogen, L7528) for 10 min at 37°C. After three washes with fresh medium, the cells were analysed by fluorescence microscopy.

### 2.29 LTR uptake in HUVECs

The treated HUVECs were incubated with a LTR fluorescent probe (50 nM, Invitrogen, L12492) for 2 h at 37°C and the excess dye was washed. Then, the cells were examined and imaged using a microscope (Olympus, Japan).

### 2.30 Lysosome isolation

Lysosomal fractions from tissues and cells were enriched and purified using the Lysosome Isolation Kit (Abcam, ab234047) by differential centrifugation followed by density gradient centrifugation.^46^, ^47^ Briefly, samples were homogenised in ice‐cold lysosome isolation buffer for 2 min. The supernatants were collected by centrifugation at 500 × *g* and layered onto a discontinuous density gradient. Lysosomes were then isolated using ultracentrifugation at 145 000 × *g* for 2 h, after which the lysosomal fraction was obtained for further analysis.

### 2.31 Experimental design

To explore the roles of NKAα1 in diabetes‐related endothelial dysfunction, this study investigated the effects of high‐glucose (HG) exposure on mouse aortae and ECs as well as HFD‐induced diabetes in WT and NKAα1^+/−^ mice. Protein and mRNA levels of NKAα1 were measured and NKA activity was also evaluated in these tissues and cells to determine the impact of diabetic conditions on NKAα1 function. EDR was tested in aortic rings from WT, NKAα1^+/−^, and NKAα1‐overexpressing mice. SNP was used to evaluate endothelium‐independent relaxation.

Oxidative/nitrative stress markers, including superoxide, NO_x_, and nitrotyrosine, were measured in HG/HFD‐induced diabetic mouse aortae and ECs. EC viability, proliferation, migration, and tube formation were measured following NKAα1 overexpression or knockdown. The effects of NKAα1 on EC apoptosis were assessed through TUNEL assays.

To investigate the role of NKAα1 in ferroptosis, ferroptosis markers, including Fe^2^⁺ levels (FerroOrange staining), GSH levels, PTGS2 mRNA, Fth1 mRNA, ACSL4, and GPX4 protein levels, were measured. Autophagy‐related protein expression, including LC3II/LC3I ratio and p62, was examined in HG/HFD‐exposed ECs and mouse aortae. The localisation of ACSL4 within lysosomes was analysed to assess lysosomal degradation efficiency. LMP was detected via double immunostaining with LGALS3 and LAMP1, and lysosomal pH changes were assessed with LysoTracker Red staining.

Eventually, a small library of 379 natural compounds was screened to identify potential NKAα1 activators. Hamaudol was identified and examined for its effects on NKAα1 expression, activity, and translocation in diabetic mouse aortae. After 6 weeks of normal diet (ND) or HFD, mice were subjected to intraperitoneal injection of Hamaudol (30 mg/kg) or sterile saline for the next 6 weeks under ND or HFD conditions.^48^ HUVECs were preincubated with Hamaudol (5 µM) for 30 min and were incubated with HG/HF for addition 24 h. The effects of Hamaudol on EDR, ferroptosis markers, oxidative/nitrative stress, ACSL4 and Cathepsin B expression, and autophagy were assessed to elucidate its therapeutic potential.

### 2.32 Statistical analysis

Results are presented as mean ± standard deviation (SD). In vitro experiments included at least three or four independent biological and technical replicates, while in vivo experiments had six replicates. Data analysis and graphing were performed using GraphPad Prism. Differences between two groups were assessed using an unpaired *t*‐test, and multiple treatment comparisons were analysed with ANOVA followed by the Bonferroni post hoc test. A *p*‐value < .05 was considered statistically significant.

## 3 RESULTS

### 3.1 Role of NKAα1 in diabetes‐elicited endothelial dysfunction

**FIGURE 1:**
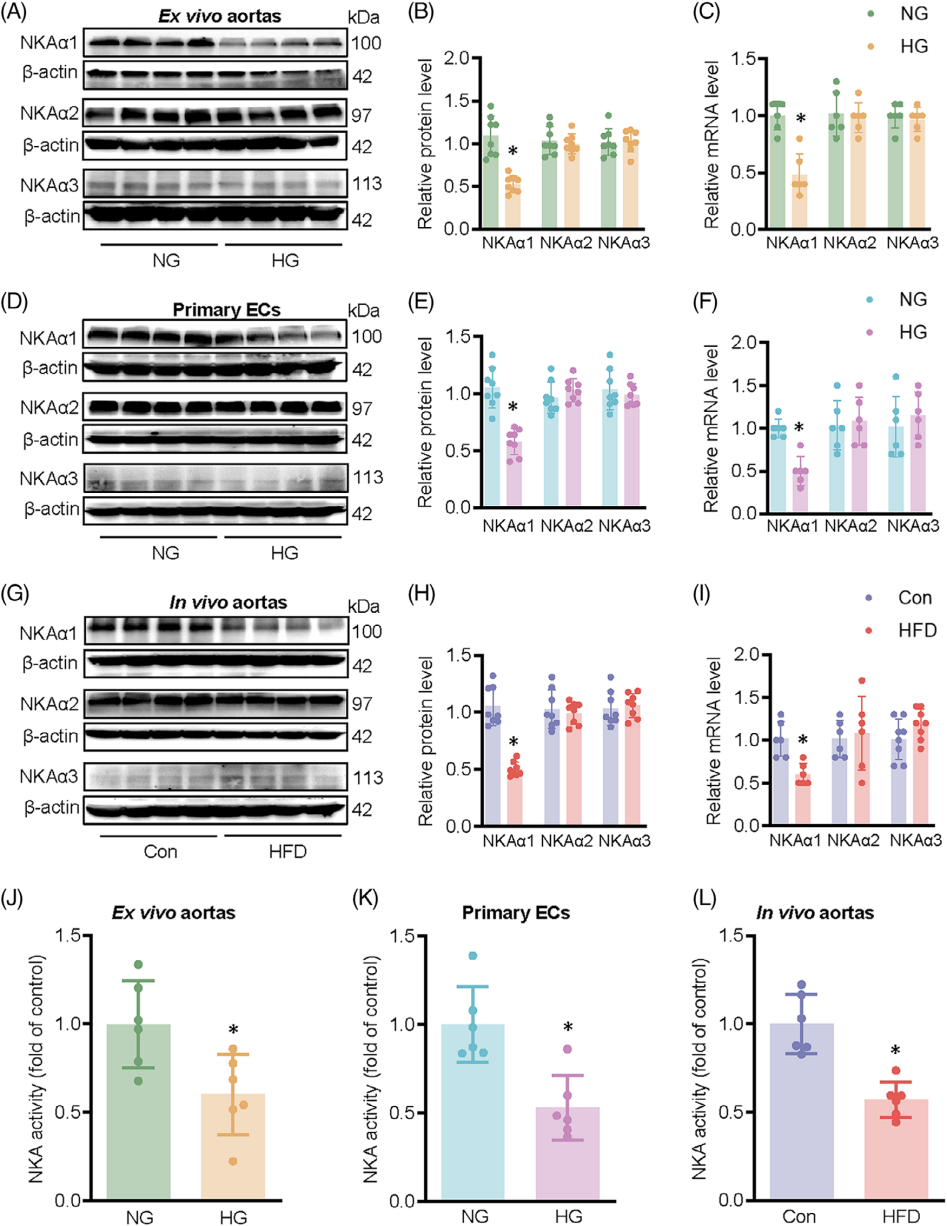
Expression of NKAα isoforms and NKA activity. (A, B) Protein expression of NKAα1, NKAα2, and NKAα3 in HG‐incubated mouse aortae. *n* = 8. (C) Relative mRNA level of NKAα1, NKAα2, and NKAα3 in HG‐incubated mouse aortae. *n* = 6. (D, E) Protein expression of NKAα1, NKAα2, and NKAα3 in HG‐incubated primary ECs. *n* = 8. (F) Relative mRNA level of NKAα1, NKAα2, and NKAα3 in HG‐incubated primary ECs. *n* = 6. (G, H) Protein expression of NKAα1, NKAα2, and NKAα3 in HFD‐induced mouse aortae. *n* = 8. (I) Relative mRNA levels of NKAα1, NKAα2, and NKAα3 in HFD‐induced mouse aortae. *n* = 6. (J) NKA activity in HG‐incubated mouse aortae. *n* = 6. (K) NKA activity in HG‐incubated primary ECs. *n* = 6. (L) NKA activity in HFD‐induced mouse aortae. *n* = 6. *p < .05 vs. normal glucose (NG) or Control (Con).

HG exposure obviously reduced the protein and mRNA expression levels of NKAα1 (Figure 1A–C), accompanied by a decreased NKA activity in mouse aortae (Figure 1J). To detect the expression and distribution of NKAα1 in human aortae, we used the online database HUMAN PROTEIN ATLAS (https://www.proteinatlas.org/) to compare the differences in NKAα1 expression levels in human aortae. It is shown that NKAα1 is mainly expressed in ECs of human aortae (Figure S1A and B). Primary cell experiments showed that NKA1 was mainly expressed in ECs, and high glucose did not affect the expression of NKA1 in vascular smooth muscle cells and vascular fibroblasts (Figure S1C). Thus, we focused on studying the role of NKAα1 in diabetes‐elicited endothelial dysfunction. Exposure to HG resulted in a marked decrease in both protein and mRNA levels of NKAα1 in ECs (Figure 1D–F), as well as disrupted NKA activity (Figure 1K). These changes were also detectable in HFD‐fed mice (Figure 1G–I and L). Interestingly, the translational and transcriptional levels of NKAα2 and NKAα3 remain unchanged in HG‐challenged aortae and ECs, as well as in aortae from HFD mice (Figure 1A–I). Overall, these results indicate that NKAα1 expression and activity are significantly impaired in endothelial dysfunction associated with diabetes.

**FIGURE 2:**
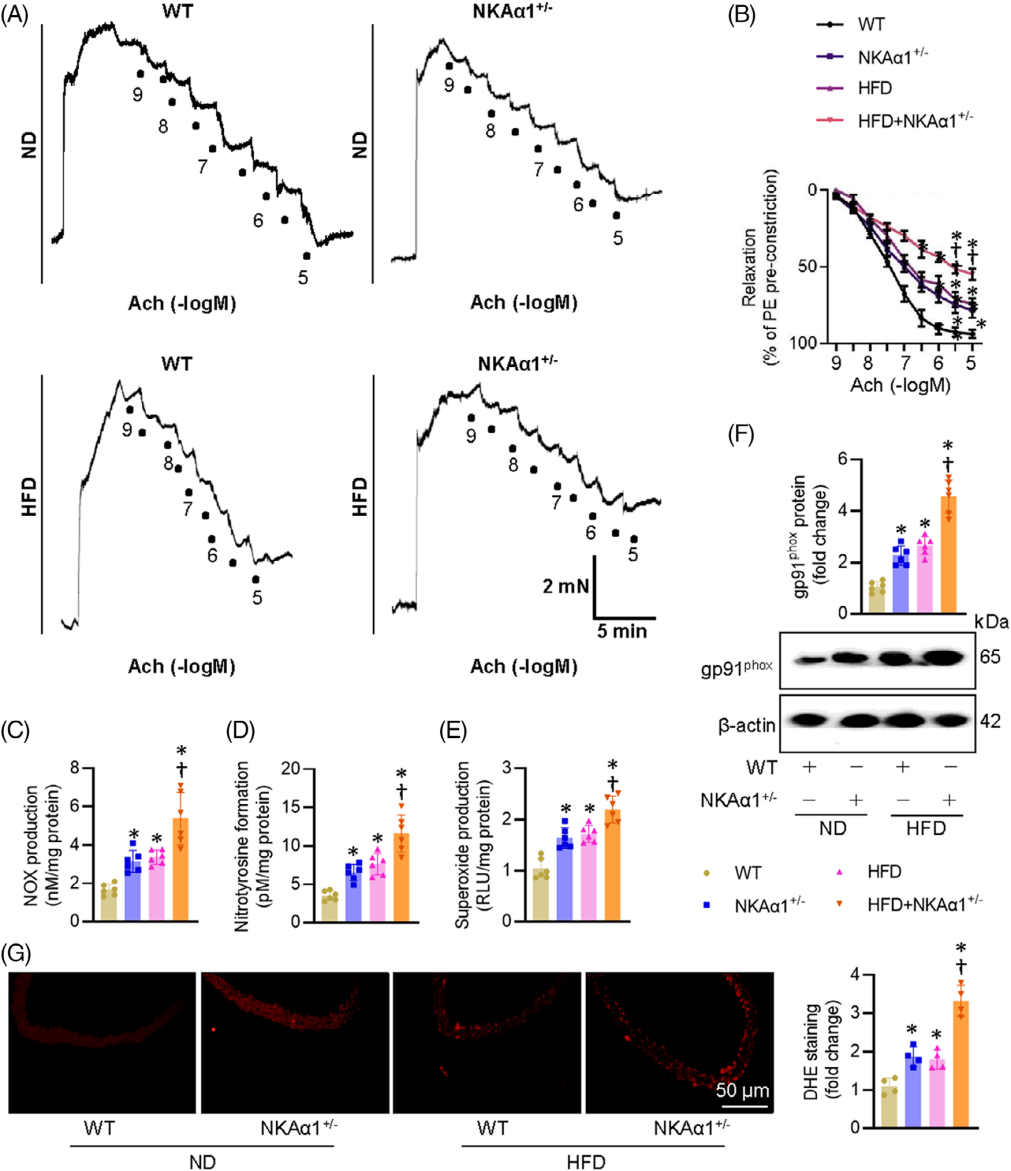
Effects of NKAα1 loss on EDR and oxidative/nitrative stresses in aortic segments. (A, B) Deficiency of NKAα1 further aggravated EDR in diabetic mice. (C) NO_x_ production. *n* = 6. (D) Nitrotyrosine formation. *n* = 6. (E) Superoxide production. *n* = 6. (F) Representative blot and quantitative analysis of gp91^phox^ protein. *n* = 4. (G) Representative image and quantitative analysis of DHE staining. *n* = 4. Scale bar, 50 µm. *p < .05 vs. Wild‐type (WT). †*p* < .05 vs. HFD.

The aortic rings of heterozygous NKAα1^+/−^ mice exhibited reduced endothelium‐dependent relaxation (EDR) in comparison with those in wild‐type (WT) mice (Figures 2A and S2A). Diabetic aortic segments showed higher levels of NO_x_, nitrotyrosine, and superoxide anions, this was further aggravated in NKAα1^+/−^ mice fed by a HFD (Figure 2B–D). Immunoblotting for gp91^phox^ and DHE staining showed increased oxidative stress in both NKAα1^+/−^ mice and diabetic mice, with these changes becoming more pronounced in HFD‐fed NKAα1*^+/−^* mice (Figure 2E–G). Conversely, EC‐specific overexpression of NKAα1 via adeno‐associated virus (AAV) ameliorated EDR in HFD mice by suppressing oxidative and nitrative stresses (Figure S3A–G). Intriguingly, the addition of the NO donor sodium nitroprusside (SNP), resulted in full vascular relaxation in all groups (Figure S4A and B). We then moved to assess the potential role of NKAα1 in EDR of ex vivo aortae incubated by HG. In line with in vivo results, EDR in HG‐exposed aortae was obviously reduced, this was further declined in mouse aortae from NKAα1 knockdown mice (Figure S5A). SNP‐induced vascular relaxation responses were not altered by loss of NKAα1 (Figure S5B). In addition, the production of NO_x_, nitrotyrosine, and superoxide anions tended to be higher in HG‐exposed ex vivo aortae, an effect that was further amplified in NKAα1‐deficient aortae (Figure S5C–E). By contrast, overexpression of endothelial NKAα1 by AAV5‐TIE1‐NKAα1 reversed these effects (Figure S5F–J). HFD mice showed higher body weight, increased levels of fasting blood glucose (FBG), upregulated serum levels of insulin, total cholesterol, and triglycerides compared to control mice (Tables S3 and S4).

### 3.2 Role of NKAα1 in HG/HF‐elicited endothelial cell injury

**FIGURE 3:**
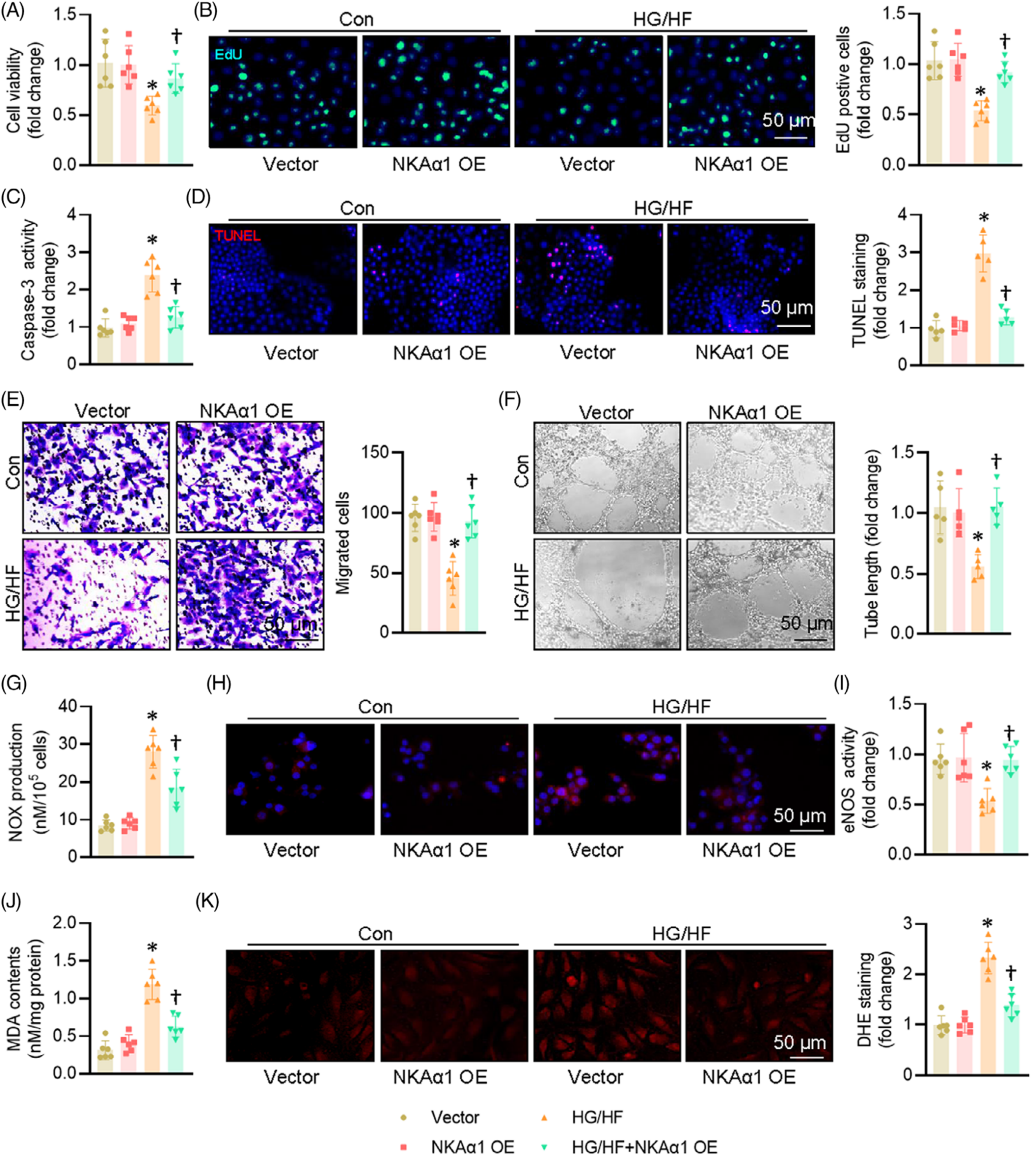
Effects of NKAα1 overexpression on HG/HF‐evoked EC injury in vitro. (A) CCK‐8 assay. *n* = 6. (B) Cell viability assessed EdU incorporation assay. *n* = 6. Scale bar is 50 µm. (C) Caspase‐3 activity. *n* = 6. (D) Cell apoptosis assessed by TUNEL analysis. *n* = 5. Scale bar, 50 µm. (E) The migration of HUVECs. *n* = 6. Scale bar is 50 µm. (F) The angiogenesis of HUVECs. *n* = 5. Scale bar is 50 µm. (G) NO_x_ production. *n* = 6. (H) Representative immunofluorescence staining of 3‐nitrotyrosine. *n* = 6. Scale bar is 50 µm. (I) eNOS activity. *n* = 6. (J) MDA contents. *n* = 6. (K) DHE staining. *n* = 6. Scale bar is 50 µm. *p < .05 vs. Vector. †*p* < .05 vs. HG/HF.

Compared with the normal glucose (NG) group, the cell viability of ECs was obviously decreased after HG/HF exposure, an effect that was partially reversed by overexpression of NKAα1 (Figures 2A and S6A). Overexpression of NKAα1 increased the proportion of EdU‐positive cells in the context of HG/HF (Figure 3B). HG/HF caused an obvious increase in caspase 3 activity, which was attenuated by NKAα1 overexpression (Figure 3C). The TUNEL‐positive cells were higher in HG/HF‐incubated cells, whereas NKAα1 overexpression reversed this phenomenon (Figure 3D). Upregulation of NKAα1 led to increased number of migrated ECs under HG/HF conditions (Figure 3E). Additionally, NKAα1 overexpression significantly abrogated HG/HF‐impaired tube formation (Figure 3F). HG/HF caused the overproduction of NO_x_ (Figure 3G) and nitrotyrosine (Figure 3H), while inhibiting the activity of eNOS in ECs (Figure 3I), effects that were mitigated by NKAα1 overexpression. Excessive formation of MDA and superoxide anions were observed in ECs upon HG/HF exposure, and these changes were reversed by NKAα1 overexpression (Figure 3J and K). Paralleling, the protein levels of IL‐1β, MCP‐1, TNF‐α, and VCAM‐1 were significantly enhanced in HG/HF‐exposed ECs, and NKAα1 overexpression eliminated these changes (Figure S6B). Similar results were also observed by real‐time PCR assays (Figure S6C). On the contrary, silencing NKAα1 further worsened the detrimental actions of HG/HF on cell viability, apoptosis, migration, tube formation, oxidative stress, and inflammation in ECs (Figures S6D–F and S7).

### 3.3 Impaired NKAα1 function results in EC ferroptosis in vitro and in vivo

**FIGURE 4:**
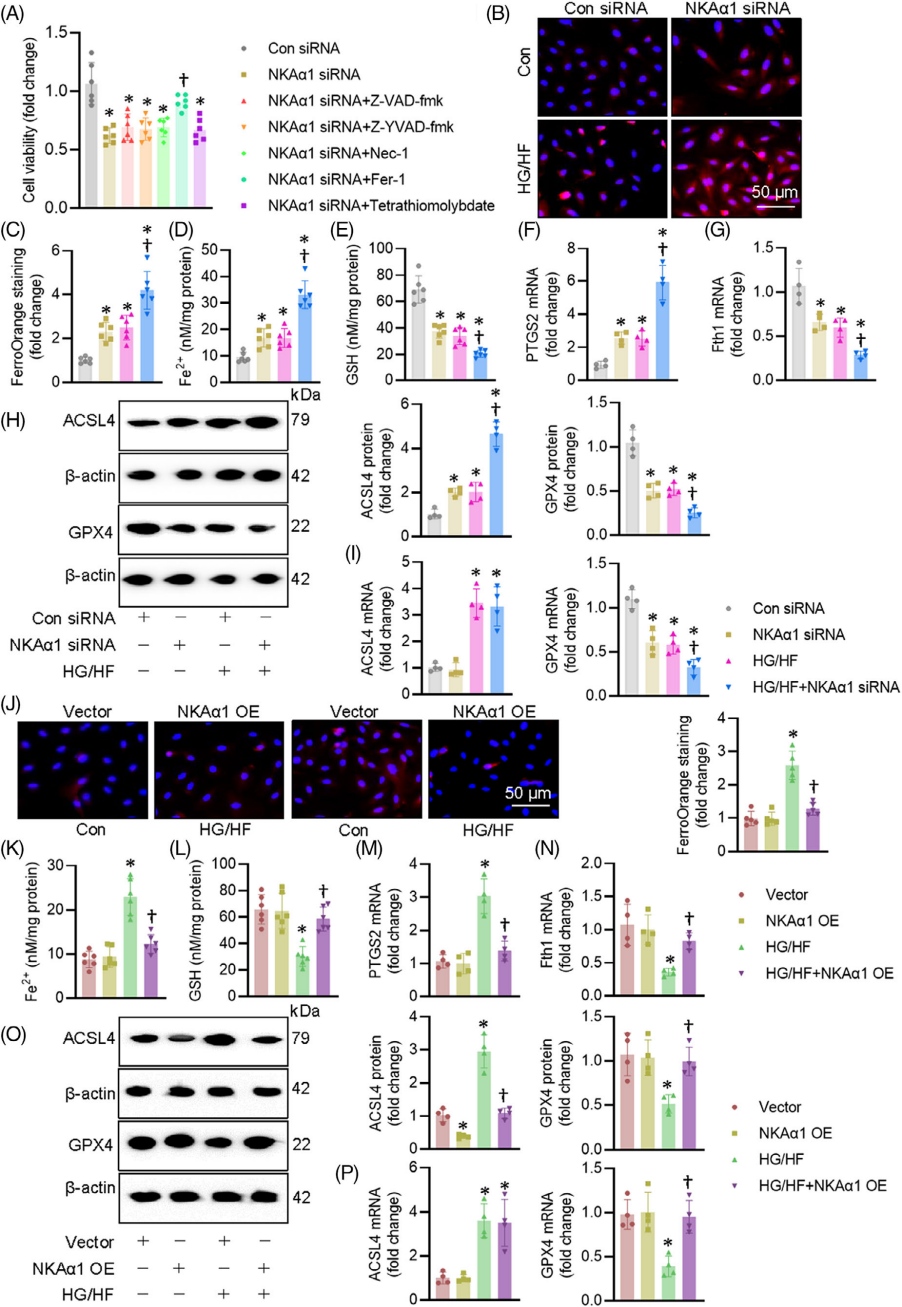
Effects of NKAα1 on HG/HF‐evoked ferroptosis in ECs. (A) CCK‐8 assay. *n* = 6. (B, C) Effects of NKAα1 knockdown on HG/HF‐evoked ferroptosis in ECs, as evidenced by FerroOrange staining. Scale bar is 50 µm. (D) Effects of NKAα1 knockdown on Fe^2+^ contents. *n* = 6. (E) Effects of NKAα1 knockdown on GSH contents. *n* = 6. (F) Effects of NKAα1 knockdown on PTGS2 mRNA level. *n* = 4. (G) Effects of NKAα1 knockdown Fth1 mRNA level. *n* = 4. (H) Effects of NKAα1 knockdown on the protein expression of ACSL4 and GPX4. *n* = 4. (I) Effects of NKAα1 knockdown on the mRNA levels of ACSL4 and GPX4. *n* = 4. (J) Effects of NKAα1 overexpression on HG/HF‐evoked ferroptosis in ECs, as evidenced by FerroOrange staining. *n* = 6. Scale bar is 50 µm. (K) Effects of NKAα1 overexpression on Fe^2+^ contents. *n* = 6. (L) Effects of NKAα1 overexpression on GSH contents. *n* = 6. (M) Effects of NKAα1 overexpression on PTGS2 mRNA. *n* = 4. (*N*) Effects of NKAα1 overexpression on Fth1 mRNA level. *n* = 4. (O) Effects of NKAα1 overexpression on the proteins of ACSL4 and GPX4. *n* = 4. (P) Effects of NKAα1 overexpression on the mRNA levels of ACSL4 and GPX4. *p < .05 vs. Con siRNA or Vector. †*p* < .05 vs. HG/HF.

We then examined how NKAα1 malfunction contributed to EC injury induced by HG/HF. The suppressive effects of NKAα1 knockdown on the cell viability were significantly mitigated by treatment a ferroptosis inhibitor Fer‐1, but not by Z‐YVAD‐fmk, Z‐VAD‐fmk, Nec‐1, or Tetrathiomolybdate, which are well‐known inhibitors of apoptosis, pyroptosis, necrosis, and cuproptosis, respectively (Figure 4A). This indicates that NKAα1 impairment may be related to ferroptosis in ECs. Therefore, ferroptosis‐related indicators were tested in ECs. As anticipated, HG/HF induced overproduction of Fe^2+^, as indicated by FerroOrange staining and Fe^2+^ levels in ECs, such effects were aggravated by knocking down NKAα1 (Figure 4B–D). Knockdown of NKAα1 exacerbated HG/HF‐induced decreases in the contents of GSH (Figure 4E). Supportively, HG/HF stimulation elevated the mRNA level of PTGS2 and suppressed the transcriptional level of ferritin heavy chain1 (Fth1) in ECs, an observation that was intensified by silencing NKAα1 (Figure 4F and G). Western blot analysis showed that NKAα1 knockdown further disrupted the actions of HG/HF on the protein expression levels of ACSL4 (a proferroptosis protein) and GPX4 (an antiferroptosis protein) (Figures 4H and S8A). NKAα1 knockdown significantly enhanced the HG/HF‐induced downregulation of GPX4 mRNA, but not ACSL4 mRNA (Figure 4I). Although NKAα1 overexpression (Figure S8B) did not affect the mRNA level of ACSL4, it is sufficient to attenuate HG/HF‐induced ferroptosis in ECs (Figure 4J–O). Overexpression of NKAα1 reduced the levels of long‐chain PUFA‐containing phospholipids, as manifested by targeted lipidomic analysis (Figure S9A). In support, Erastin, a known ferroptosis inducer, abolished the protective effects of NKAα1 overexpression on HG/HF‐induced apoptosis, oxidative/nitrative stresses, rather than inflammatory response in ECs (Figure S10A–D). Taken together, we found that decreased NKAα1 activity exacerbated HG/HF‐induced ferroptosis, indicating a central role of NKAα1 in the ferroptosis induced by HG/HF.

**FIGURE 5:**
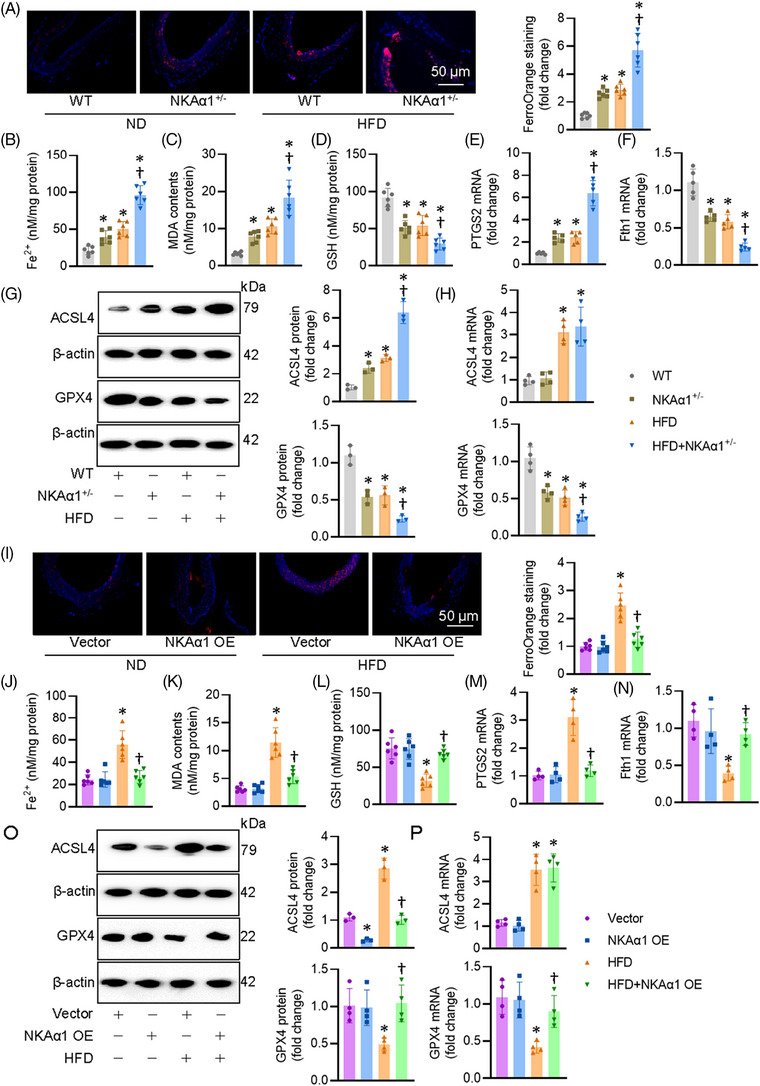
Effects of NKAα1 on HFD‐evoked ferroptosis in mouse aortae. (A) Effects of NKAα1 loss on HFD‐evoked ferroptosis in mouse aortae, as evidenced by FerroOrange staining. Scale bar is 50 µm. (B) Effects of NKAα1 knockdown on Fe^2+^ contents in mouse aortae. *n* = 6. (C) Effects of NKAα1 knockdown on MDA contents in mouse aortae. *n* = 6. (D) Effects of NKAα1 knockdown on GSH contents in mouse aortae. *n* = 6. (E) Effects of NKAα1 knockdown on PTGS2mRNA level in mouse aortae. *n* = 5. (F) Effects of NKAα1 knockdown on Fth1mRNA level in mouse aortae. *n* = 5. (G) Effects of NKAα1 knockdown on the protein expression levels of ACSL4 and GPX4 in mouse aortae. *n* = 3. (H) Effects of NKAα1 knockdown on the transcriptional levels of ACSL4 and GPX4 in mouse aortae. *n* = 4. (I) Effects of endothelial cell‐specific overexpression of NKAα1 on HFD‐evoked ferroptosis in mouse aortae, as evidenced by FerroOrange staining. Scale bar is 50 µm. (J) Effects of endothelial cell‐specific overexpression of NKAα1 on Fe^2+^ contents in mouse aortae. *n* = 6. (K) Effects of endothelial cell‐specific overexpression of NKAα1 on MDA contents in mouse aortae. *n* = 6. (L) Effects of endothelial cell‐specific overexpression of NKAα1 on GSH contents in mouse aortae. *n* = 6. (M) Effects of endothelial cell‐specific overexpression of NKAα1 on the mRNA level of PTGS2 in mouse aortae. *n* = 4. (N) Effects of endothelial cell‐specific overexpression of NKAα1 on the mRNA level of Fth1 in mouse aortae. *n* = 4. (O) Effects of endothelial cell‐specific overexpression of NKAα1 on the protein expression of ACSL4 and GPX4 in mouse aortae. *n* = 3 or 4. (P) Effects of endothelial‐specific overexpression of NKAα1 on the mRNA levels of ACSL4 and GPX4 in mouse aortae. *n* = 4. *p < .05 vs. WT or Vector. †*p* < .05 vs. HFD.

In consistence with cellular results, NKAα1‐deficient mice were predisposed to ferroptosis in aortae after HFD feeding (Figures 5A–H and S8C), while endothelial specific overexpression of NKAα1 increased the resistance of mouse aortae to HFD‐induced ferroptosis (Figures 5I–O and S8D). Similar results were obtained from primary ECs in WT and NKAα1 heterozygous mice (Figure S11A–D). To further confirm the fundamental role of ferroptosis in NKAα1 deletion in vascular endothelial dysfunction in diabetes, we used Fer‐1 and Erastin in mouse aortae. As expected, administration of a ferroptosis inhibitor Fer‐1 restored impaired EDR in aortae from NKAα1^+/−^ mice (Figure S12A), in conjunction with decreased contents of superoxide, NO_x_, and nitrotyrosine in aortic tissues (Figure S12B–D). Meanwhile, the benefits of NKAα1 overexpression in EDR were eliminated by the presence of Erastin, an inducer of ferroptosis (Figure S12E). Erastin treatment prevented the suppressive actions of NKAα1 overexpression on oxidative/nitrative stresses in aortae from HFD‐fed mice (Figure S12F–H).

### 3.4 Role of NKAα1 in diabetes‐triggered impairment in lysosome biosynthesis and function

**FIGURE 6:**
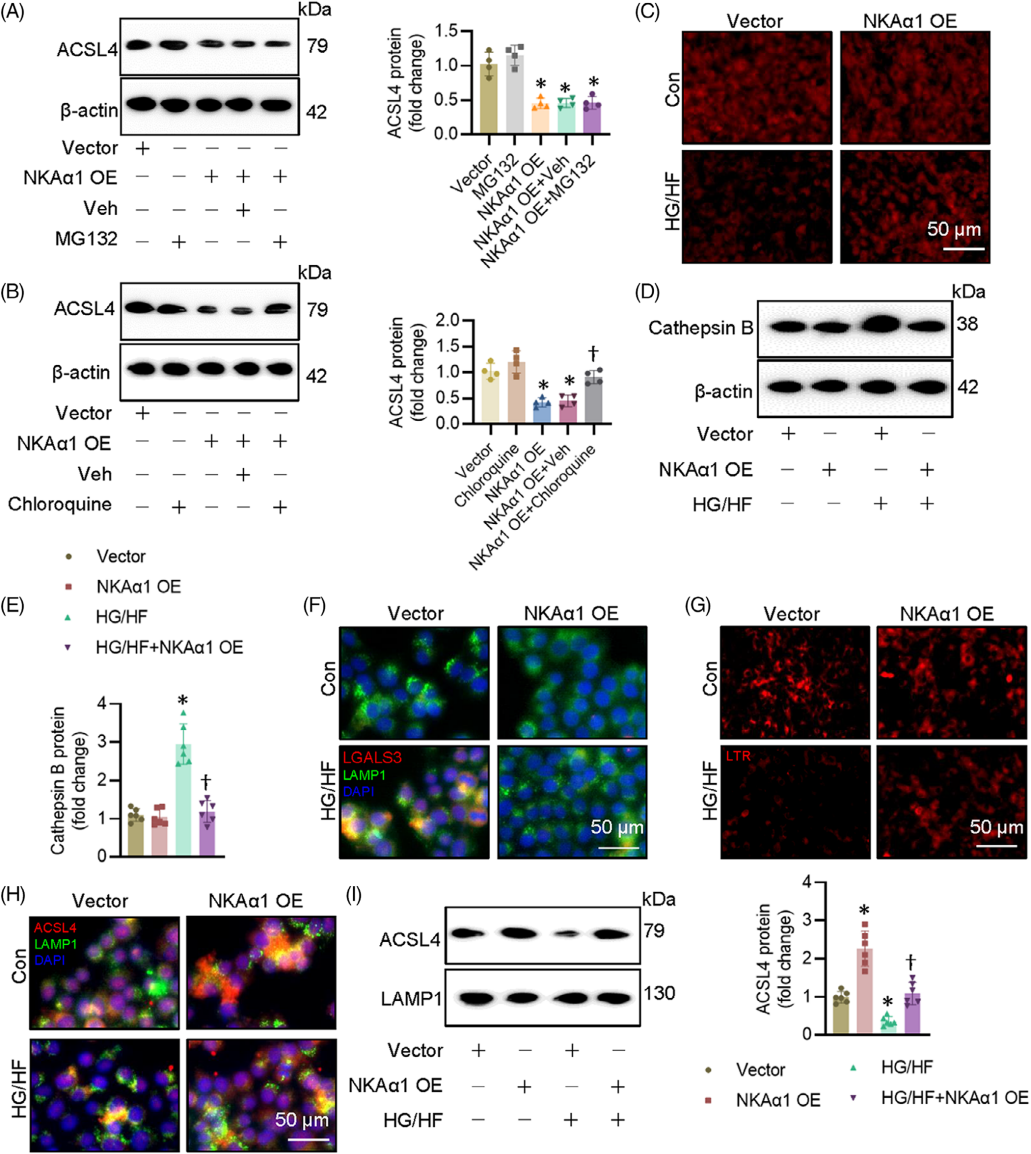
Effects of NKAα1 on the lysosome function in ECs. (A) The proteasome inhibitors MG‐132 did not affect NKAα1 overexpression‐induced ACSL4 degradation. *n* = 3. (B) The lysosome inhibitor chloroquine dramatically reversed the degradation of ACSL4 induced by NKAα1 overexpression. *n* = 4. (C) NKAα1 overexpression conserved lysosome integrity in HUVECs, as assessed by Lysotracker DND‐99 staining. Lysotracker DND‐99, a lysosomotropic dye, was used to mark lysosomes in the context of acidic pH. A decrease in its fluorescence intensity indicates disruptions in lysosomal function, integrity, or quantity. Scale bar is 50 µm. (D, E) NKAα1 overexpression prevented cathepsin B release in HUVECs. *n* = 6. (F) Immunofluorescence double staining of LGALS3 and LAMP1 in HUVECs. Scale bar is 50 µm. (G) LysoTracker Red (LTR) fluorescent probe was used to detect lysosome membrane permeabilisation in HUVECs. Scale bar is 50 µm. (H) Immunofluorescence double staining of ACSL4 and LAMP1 in HUVECs. *n* = 6. Scale bar is 50 µm. (I) Representative blots showing the abundance of NKAα1 in the lysosome. *n* = 6. *p < .05 vs. Vector. †*p* < .05 vs. or NKAα1 OE+Veh or HG/HF.

NKAα1 inversely affected the protein expression of ACSL4 without influencing its mRNA level, indicating the posttranslational modification might mediate the actions of NKAα1 on ACSL4. It is well established that the uniquitin‐proteasome system and the lysosome system can mediate the degradation of intercellular protein. To determine which pathway is responsible for NKAα1‐induced ACSL4 degradation, we treated ECs with proteasome and lysosome inhibitors. NKAα1‐induced downregulation of ACSL4 was significantly attenuated by chloroquine, a lysosome inhibitor, but not by a proteasome inhibitor MG132 (Figure 6A and B). This suggests that the lysosome system may be involved in NKAα1 knockdown‐induced upregulation of ACSL4. As depicted in Figure 6C, HG/HF markedly decreased lysotracker staining, but this was prevented by NKAα1 overexpression. We also observed that NKAα1 overexpression reduced cathepsin B accumulation in ECs upon HG/HF exposure (Figure 6D and E), suggesting that NKAα1 overexpression is able to prevent the release of cathepsin B from lysosomes. The lysosome membrane permeabilisation (LMP) is reported to contribute to impaired autophagy degradation. To investigate whether LMP occurs in ECs following HG/HF exposure, double immunostaining for LGALS3 and LAMP1 was performed. The results showed an increase in LGALS3‐positive dots in HG/HF‐exposed cells, with a marked colocalisation of LGALS3 and LAMP1 puncta. These changes were significantly reversed by NKAα1 overexpression (Figure 6F). To further assess LMP, lysosomal pH was evaluated using the LysoTracker Red (LTR) fluorescent probe, which selectively labels acidic organelles in live cells. LTR fluorescence was reduced in ECs after HG/HF exposure, but this effect was reversed upon NKAα1 overexpression (Figure 6G). These findings suggest that LMP may contribute to the reduced autophagic degradative capacity in ECs under HG/HF conditions, a dysfunction that can be corrected by NKAα1 overexpression. To corroborate such observations, we measured the protein expression of ACSL4 in the lysosome. Both immunostaining and immunoblotting showed that NKAα1 overexpression prevented the effects of HG/HF on the location of ACSL4 in the lysosome (Figure 6H and I). Inversely, silencing NKAα1 further decreased the accumulation of ACSL4 in the lysosome of ECs challenged by HG/HF (Figure S13A). The lysosome inhibitor chloroquine prevented the actions of NKAα1 overexpression on cell apoptosis, oxidative/nitrative stresses, and ferroptosis in ECs after HG/HF challenge (Figure S14A–G). The antagonistic effects of NKAα1 overexpression on oxidative/nitrative stresses and EDR impairment were also impeded after administration of chloroquine (Figure S14H–M).

### 3.5 NKAα1 deficiency led to autophagy dysfunction in diabetes‐induced endothelial injury

**FIGURE 7:**
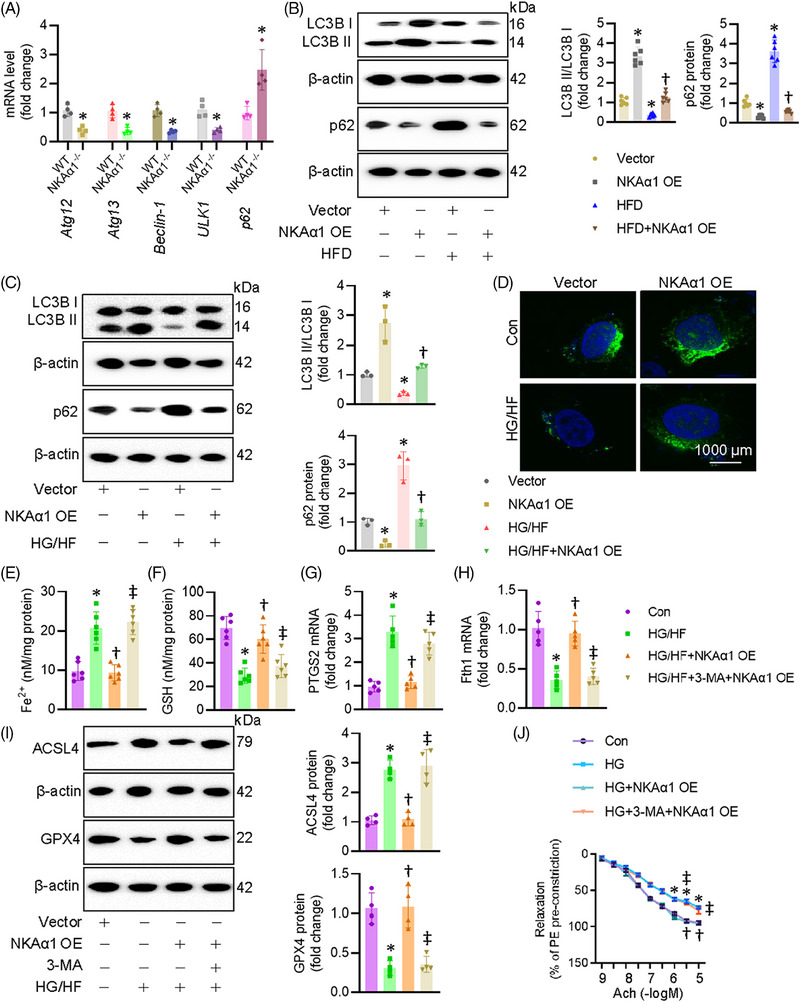
Effects of NKAα1 on the function of autophagy in ECs. (A) Relative mRNA levels of Atg12, Atg13, Beclin‐1, ULK1, and p62. (B) Representative blot of LC3B and p62 in mouse aortae. (C) Representative blot of LC3B and p62 in HUVECs. (D) Autophagosome detection by EGFP‐LC3 plasmid transfection. *n* = 6. Scale bar is 1000 µm. (E) Fe^2+^ contents. *n* = 6. (F) GSH contents. *n* = 6. (G) Relative PTGS2 mRNA level. *n* = 5. (H) Relative mRNA level of Fth1. *n* = 5. (I) Representative blots of ACSL4 and GPX4 in HUVECs. *n* = 4. (J) Inhibition of autophagy with 3‐MA abolished the effects of NKAα1 on EDR. *n* = 6. *p < .05 vs. Vector or Con. †*p* < .05 vs. or HG or HG/HF. ‡*p* < .05 vs. NKAα1 OE+HG or NKAα1 OE+HG/HF.

Autophagy is a cleansing and reparative process that delivers cellular cargoes to lysosomes for degradation. This process begins with the formation of autophagosomes, requiring class III phosphoinositide 3‐kinase (PI3K) Vps34 to interact with beclin‐1. After membrane elongation, autophagosomes fuse with lysosomes, allowing lysosomal hydrolases to degrade the waste in cells. We previously demonstrated that NKAα1 deficiency resulted in the disruption of autophagy, a critical event in nonalcoholic fatty liver disease, brain ischemic injury, and Parkinson's disease. Based on this, we hypothesised that malfunction of NKAα1 might cause the disordered autophagy‐lysosome pathway in diabetic endothelial dysfunction. As shown in Figure 7A, the mRNA levels of autophagy‐related genes, including Atg12, Atg13, beclin‐1, ULK1 and p62, were significantly altered in mouse aortae after HFD feeding. Immunoblotting results showed that the aortae from HFD mice exhibited lower expression of LC3II/LC3I ratio and higher expression of p62, both of which were reversed by endothelial‐specific overexpression of NKAα1 (Figure 7B). The mRNA level of beclin‐1 was lower, while the transcriptional level of p62 was higher in isolated ECs from NKAα1 heterozygous mice than those from WT mice (Figure S11E and F). In a similar way, overexpression of NKAα1 restored LC3II/LC3I ratio and downregulated p62 protein expression in ECs exposed to HG/HF (Figure 7C). In line with this, NKAα1 overexpression increased the number of LC3 puncta‐positive cells and reversed the reduction in LC3 puncta observed in HG/HF‐treated ECs (Figure 7D). Blockaded of autophagy by 3‐MA abolished the suppressive actions of NKAα1 overexpression on ferroptosis (Figure 7E–I) and cell injury induced by HG/HF (Figure S15A–D). Most importantly, the vasodilation effects of NKAα1 overexpression were significantly weakened when the autophagy was inhibited by 3‐MA (Figure 7J). In compliance with these findings, 3‐MA pretreatment overtly abolished the protective effects of NKAα1 on oxidative/nitrative stresses in HG‐incubated mouse aortae (Figure S16A–D).

NKAα1 has been shown to interact with specific cell signalling pathways, including the mTOR and AMPK pathways, both of which play key roles in regulating autophagy.^17^, ^49^ Thus, we examined whether NKAα1 induced autophagy processes in ECs by regulating the AMPK/mTOR pathway. Consistent with our previous studies,^17^, ^49^ we found that NKAα1 interacted with AMPKα1, and this complex was higher in HG‐induced ECs (Figure S17A). NKAα1 overexpression induced AMPK phosphorylation, attenuated the increased phosphorylation of mTOR (p‐mTOR; Ser2448) and reversed the decreased phosphorylation of ULK1 (p‐ULK1, Ser555) and AMPK (p‐AMPK, Thr172) in HG‐challenged ECs (Figure S17B). These findings suggest that NKAα1/AMPKα1 interaction could exert a regulatory effect on the autophagy process in ECs, likely through the AMPK/mTOR/ULK1 signalling pathway.

### 3.6 Hamaudol is identified as an activator of NKAα1 with a protection against diabetes‐induced endothelial dysfunction

**FIGURE 8:**
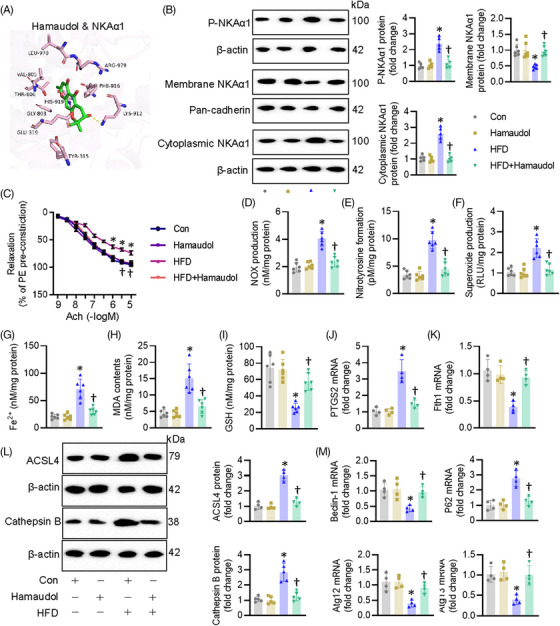
Hamaudol is identified as a potential activator of NKAα1 with endothelial benefits in HFD mice. (A) Molecular docking showing the direct binding of Hamaudol to NKAα1. (B) Hamaudol reduced the phosphorylation and cytoplasmic translocation in mouse aortae. *n* = 5. (C) Hamaudol improved EDR impairment in HFD mice. *n* = 6. (D) NO_x_ production. *n* = 6. (E) Nitrotyrosine formation. *n* = 6. (F) Superoxide production. *n* = 6. (G) Fe^2+^ contents. *n* = 6. (H) MDA contents. *n* = 6. (I) GSH contents. *n* = 6. (J) Relative mRNA level of PTGS2. *n* = 4. (K) Relative mRNA level of Fth1. *n* = 4. (L) Representative blot and quantitative analysis of ACSL4 and Cathepsin B. *n* = 4. (M) Relative mRNA levels of Beclin‐1, p62, Atg12, and Atg13. *n* = 4. *p < .05 vs. Con. †*p* < .05 vs. HFD.

Based on the data above, overexpression of NKAα1 appears to be a feasible therapeutic strategy for diabetes‐evoked endothelial dysfunction. For this reason, we sought to screen a potential activator of NKAα1 from a small library containing 379 natural products. Our screening results showed that Hamaudol displayed the strongest abilities to enhance NKAα1 activity (Table S5). Molecular docking results confirmed the direct direction interaction between Hamaudol and NKAα1, with a binding energy of −5.64 KJ/mol (Figure S18A). More specifically, 10 hydrogen bonds were formed between Hamaudol and the Tyr315, Glu319, Gly803, Val805, Thr806, Lys912, Phe916, His919, Leu978, and Arg979 in the ligand‐binding domain of NKAα1 (Figure 8A). Functional results demonstrated that pretreatment with Hamaudol restored the protein expression of NKAα1 and NKA activity in ECs exposed to HG/HF (Figure S18B and C). Cellular experiments showed that Hamaudol increased EC viability, promoted the migration and tube formation of ECs under HG/HF stress (Figure S18D–G). Impaired activity of NKA is found to be its phosphorylation and cytoplasmic translocation, and this event leads to a variety of oxidative stress‐related diseases. To this end, we measured the phosphorylation levels of NKAα1 and the membrane and cytoplasmic abundance of NKAα1. Hamaudol treatment reduced NKAα1 phosphorylation and prevented its translocation from the membrane to the cytoplasm in HFD‐induced mouse aortae (Figure 8B), accompanied by restored NKA activity (Figure 8C). Furthermore, Hamaudol treatment diminished diabetes‐induced EDR impairment (Figure 8D). The contents of NO_x_, nitrotyrosine, and superoxide anions in diabetic mouse aortae were obviously downregulated by treatment with Hamaudol (Figure 8E–G). The Fe^2+^ contents and lipid peroxidation levels were markedly raised in diabetes and significantly reduced after treatment with Hamaudol (Figure 8H and I). In addition, Hamaudol supplementation retarded ferroptosis, as evidenced by GSH levels, mRNA levels of PTGS2 and Fth1 altered by the HFD (Figure 8J and K). In accordance with the results of NKAα1 overexpression, administration of Hamaudol prevented diabetes‐induced protein upregulations of ACSL4 and Cathepsin B (Figure 8L), and induced autophagy in diabetic mouse aortae (Figure 8M). Interestingly, administration of Hamaudol had no effect on the fasting blood glucose and lipid metabolism in HFD‐induced mice (Table S6). Overall, these findings suggest that Hamaudol achieved its favourable effects in the context of diabetes‐evoked endothelial cell dysfunction by targeting NKAα1 and inhibiting ferroptosis. Vascular remodelling is a well‐established pathological feature in diabetes, often characterised by endothelial dysfunction, vascular smooth muscle cell proliferation, increased collagen deposition, and thickening of the arterial walls. It is interesting to know whether Hamaudol has an effect on vascular remodelling in diabetic mice. Results showed that the lumen diameter was significantly downregulated, along with a marked increase in medial thickness and the medial thickness/lumen diameter ratio in diabetic mice in comparison with the control mice. These alterations were attenuated by administration of Hamaudol (Figure S19A–D). The mRNA levels of pro‐proliferative, propro‐fibrotic and proinflammatory genes were substantially upregulated in the aortae of HFD mice, which were largely downregulated by Hamaudol treatment (Figure S19E–G). These results indicate that Hamaudol not only mitigates endothelial dysfunction but also helps alleviate vascular remodelling processes in the context of diabetes.

## 4 DISCUSSION

**FIGURE 9:**
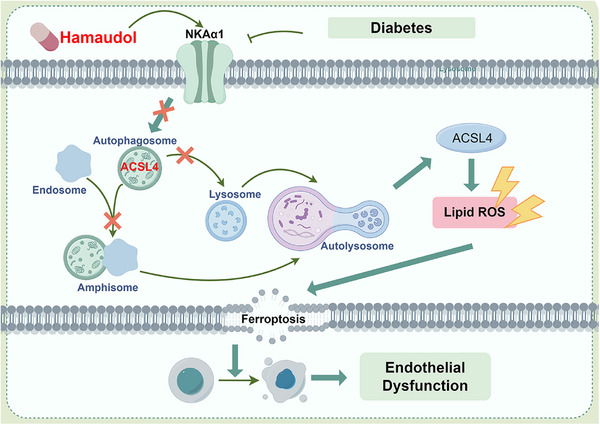
Schematic illustration showing the role and molecular mechanism of NKAα1 in diabetes‐induced endothelial dysfunction.

The present study demonstrates an important role for NKAα1 in improving diabetes‐related endothelial dysfunction induced by a HFD diet. In vitro, ex vivo and in vivo research disclosed lower NKA activity and NKAα1 expression in diabetic endothelial dysfunction. We found that NKAα1 haploinsufficiency significantly aggravated diabetes‐elicited endothelial dysfunction in mice and silencing NKAα1 worsened HG/HF‐induced impairment in EC viability, migration and angiogenesis. Endothelial cell‐specific overexpression of NKAα1 protected against such dysfunction in diabetes. Mechanistically, NKAα1 deficiency impaired the autophagy‐lysosome pathway, thereby leading to reduced ACSL4 degradation and enhanced lipid peroxidation and ferroptosis (Figure 9). Inhibition of the autophagy‐lysosome pathway blocked the protection of NKAα1 overexpression against endothelial damage in diabetes. Most importantly, from a small natural product compound library, we identified Hamaudol as a NKAα1 activator by reducing the phosphorylation and endocytosis of NKAα1, and this compound displayed similar vasoprotective benefits as overexpression of NKAα1 in vivo and in vitro. With the aid of gain‐of‐function and loss‐of‐function approaches, we demonstrated that targeting NKAα1 may be recommended as a promising avenue for the management of vascular complications associated with diabetes.

In this study, our results demonstrate that deficiency or dysfunction of NKAα1 compromises vasomotor function in the aortae of HG‐exposed and HFD‐induced diabetic mice, reflected by a significant reduction in EDR responses. These effects were aggravated by NKAα1 knockdown but ameliorated by its overexpression, suggesting that NKAα1 preservation is crucial for maintaining vasomotor tone in diabetic contexts. Given that impaired vasodilation is a hallmark of endothelial dysfunction in diabetes, our findings position NKAα1 as a potential target to restore vasomotor function. This aligns with previous reports linking NKAα1 dysfunction to cardiovascular and metabolic diseases, although our study uniquely emphasises its critical role in diabetic vasculature and suggests that enhancing NKAα1 activity could counter diabetes‐related vascular complications. The higher expression of NKAα1 in ECs could reflect the specialised role of NKAα1 in maintaining ion gradients and membrane potential within the endothelium, which is crucial for regulating endothelial cell function, vascular tone, and permeability. The aorta, being a complex tissue with multiple cell types, may show a more diluted or lower level of NKAα1 expression when averaged across all cell types. One should bear in mind that other subunits of NKA may play different roles in different types of vascular cells, which deserves further exploration in the future.

Our study extends beyond the vasodilatory role of NKAα1 by showing that NKAα1 directly supports EC viability, migration, and angiogenesis, fundamental processes for vascular repair and integrity. The observed improvements in EC function following NKAα1 overexpression and the detrimental effects of NKAα1 knockdown suggest that NKAα1 actively participates in maintaining cellular health. Mechanistically, we found that NKAα1 suppressed oxidative and nitrative stress markers, including superoxide, NO_x_, and nitrotyrosine, in HG/HF‐challenged ECs. This indicates that NKAα1 mitigates oxidative damage by possibly modulating ion gradients and membrane stability, which are critical to reduce ROS formation. These results emphasise a critical role of NKAα1 as a cellular protector against oxidative stress, a major driver of endothelial dysfunction in diabetes.

It was recently reported that impaired NKA activity is involved in the pathological process of doxorubicin‐induced cardiotoxicity^14^ and Parkinson's disease .^16^ We therefore further moved on to assess whether NKAα1 protected against diabetes‐induced endothelial dysfunction by suppressing ferroptosis. In this study, we found that overexpression of NKAα1 was sufficient to suppress HG/HF‐induced ferroptosis in ECs, while the development of ferroptosis was further aggravated in NKAα1‐dificient ECs exposed to HG/HF. Similar results were replicated in HFD mice. Our present results indicated that NKAα1 overexpression profoundly dampened diabetes‐provoked endothelial injury by suppressing ferroptosis. Under diabetic conditions, the increase in Fe^2^⁺ levels can be attributed to enhanced oxidative stress and lipid peroxidation, both of which are known to alter cellular iron homeostasis. Excessive iron in its ferrous form (Fe^2^⁺) can generate ROS through the Fenton reaction, exacerbating oxidative stress in cells exposed to diabetes. This environment often disrupts iron regulatory proteins and may increase cellular iron uptake or decrease iron storage, leading to elevated Fe^2^⁺ levels in cells. When NKAα1 is overexpressed in ECs exposed to diabetes, it likely mitigates these effects by reducing oxidative stress and stabilising iron homeostasis. NKAα1 is known to maintain cellular ion gradients and modulate intracellular signalling pathways related to oxidative stress responses. By decreasing ROS levels, NKAα1 overexpression could reduce Fe^2^⁺ accumulation, as less ROS production can decrease the dysregulation of iron metabolism. Moreover, NKAα1 may help to regulate the expression of iron transporters and storage proteins, leading to a decrease in intracellular Fe^2^⁺ levels. In summary, the increased Fe^2^⁺ levels under diabetic conditions were likely resulted from oxidative stress‐induced iron dysregulation. NKAα1 overexpression helps to reverse this effect by alleviating oxidative stress and restoring iron homeostasis, thus lowering Fe^2^⁺ levels in this context.

A plethora of proteins are postulated to contribute to the process of ferroptosis, such as antiferroptosis genes, such as SLC7A11 and GPX4, and proferroptosis genes, including PTGS2, TFRC, and ACSL4.^50^ Among them, ACSL4 has gained a widespread recognition in the development of ferroptosis since ACSL4 is able to shape cellular lipid composition by increasing the contents of long polyunsaturated ω6 fatty acids, a central event in ferroptosis.^51^, ^52^ ACSL4‐mediated regulation of ferroptosis is involved in high‐fat‐diet (HFD)‐induced cardiac remodelling and dysfunction.^53^ In this study, we found that NKAα1 overexpression enormously obliterated the protein expression of ASCL4 and the contents of long‐chain PUFA‐containing phospholipids species in ECs exposed to HG/HF. Neither NKAα1 overexpression nor NKAα1 downregulation affected the mRNA of ACSL4, indicating that NKAα1 regulated the expression ACSL4 in a manner that was dependent on the protein posttranslational modification mechanism. Further studies revealed that chloroquine, a lysosome inhibitor, prevented NKAα1‐induced downregulation of ACSL4, indicating that the disrupted lysosome system may participate in NKAα1 deficiency‐induced upregulation of ACSL4. This novel insight highlights a protective feedback mechanism where NKAα1 not only mitigates oxidative stress but also facilitates cellular clearance processes that prevent ferroptosis in diabetes. By preserving lysosomal function, NKAα1 may also prevent the release of iron from lysosomes, thus further reducing ferroptosis risk, a finding that opens new therapeutic avenues targeting lysosomal and autophagic processes in diabetes.

In our study, we observed that NKAα1 overexpression appears to stabilise lysosomes, potentially inhibiting LMP and thereby preserving lysosomal integrity. The prevention of LMP would likely reduce the release of iron from lysosomes into the cytoplasm. This stabilisation could indeed protect cells under HG stress, not only by reducing free cytosolic iron that would otherwise catalyse ROS production and promote ferroptosis but also by maintaining cellular iron homeostasis. Regarding ferritin, if LMP is blocked by NKAα1 overexpression, ferritin within lysosomes would remain stable, as it would not be subjected to lysosomal degradation. This could explain why ferritin levels are maintained, reducing the availability of free iron and thereby limiting iron‐catalysed lipid peroxidation, a key feature of ferroptosis. Thus, NKAα1 overexpression may indirectly contribute to ferroptosis inhibition by restricting iron efflux rather than only directly influencing ACSL4 degradation alone. In this context, ACSL4, an enzyme involved in synthesising proferroptotic lipids, would be less impactful if iron levels are controlled, as the substrate for lipid peroxidation would be limited. This suggests that the protective mechanism of NKAα1 might be related to reducing iron release via lysosomal stabilisation and subsequently limiting conditions conducive to ferroptosis. In summary, while our study originally focused on ACSL4 degradation as a marker for autophagic impairment, it is likely the protective role of NKAα1 may also involve the prevention of iron efflux due to stabilised lysosomes, thereby indirectly reducing ferroptosis, which needs more solid evidence.

By screening our phytochemical compound library, we found that the phytochemical Hamaudol directly binds to NKAα1 and induces its activation by suppressing the phosphorylation and endocytosis of NKAα1. Our animal and cellular results consistently disclosed that Hamaudol treatment significantly improved endothelial dysfunction under diabetic state by restoring NKAα1 protein expression and inhibiting ACSL4‐mediated ferroptosis. Interestingly, the effects of Hamaudol were specific to the functional enhancement of NKAα1, as it did not significantly alter glucose or lipid profiles in HFD mice. This specificity may allow Hamaudol to offer vascular benefits in diabetes without affecting broader metabolic parameters, making it a focused therapeutic candidate. Given the involvement in both autophagy and ferroptosis regulation of NKAα1, activating NKAα1 with compounds like Hamaudol could provide a novel approach to prevent ferroptosis and oxidative damage in diabetic vasculature. Although the benefit of Hamaudol in diabetic vascular remodelling, more studies are recommended to perform specific experiments in diabetic mouse models to evaluate vascular remodelling and cardiovascular events following Hamaudol treatment.

## 5 CONCLUSIONS

In summary, our characterisation of the autophagy‐lysosome pathway dysregulation and subsequent ferroptosis provides novel mechanistic insights into the potential role of NKAα1 deficiency in the development of endothelial cell dysfunction induced by diabetes. Our study also revealed that the phytochemical Hamaudol activated NKAα1 to ameliorate endothelial damage, making it a promising drug candidate for managing diabetes‐associated cardiovascular ailments.

Although our study provides strong evidence for the role of NKAα1 in endothelial dysfunction under diabetic conditions, several limitations should be presented in the study. Firstly, although our in vivo findings in HFD‐induced diabetic mice support solid evidence for the protective role of NKAα1 in vascular function, the results are derived from a single animal model of diabetes. It would be important to confirm these findings in other models of diabetes, such as genetically modified mice or nonobese diabetic (NOD) mice. Secondly, while our study focuses on ECs and their function, NKAα1 is also expressed in cardiomyocytes, smooth muscle cells, and its systemic effects on these tissues were not explored. Future studies could investigate whether the observed vascular benefits are solely due to endothelial NKAα1 modulation or if other cell types contribute to the overall therapeutic effects. Thirdly, future studies should investigate the potential of NKAα1 activators in clinical settings and investigate whether NKAα1 modulation could benefit other complications associated with diabetes, such as cardiomyopathy, neuropathy and nephropathy, where ferroptosis and oxidative stress are also implicated. Fourthly, NKAα1 heterozygous mice do not represent an EC‐specific knockdown model. Future studies utilising EC‐specific knockdown models (Cre‐flox technology) are needed to further dissect the role of NKAα1 specifically in ECs under diabetic context. Fifthly, in our current study, ECs were not isolated from NKAα1 heterozygous and WT mice for a direct, cell‐specific analysis of the pathways. While this approach provides valuable information about the systemic effects of NKAα1 on vascular tissues, isolating ECs would allow for more precise analysis of the pathways specifically within ECs. Future studies are required to isolate ECs from NKAα1 heterozygous and WT mice to confirm the observed functional changes at the cellular level. To refine our understanding of the specific pathways influenced by NKAα1 in endothelial cells, we plan to perform endothelial cell‐specific analyses using primary EC cultures from NKAα1 heterozygous and WT mice. This will allow us to confirm whether apoptosis, ferroptosis, lysosome biosynthesis, and autophagy are indeed modulated by NKAα1 specifically in endothelial cells. Additionally, further research is needed to understand the precise signalling pathways that link NKAα1 to autophagy and lysosomal function in diverse types of vascular cells.

## AUTHOR CONTRIBUTIONS

Xue‐Xue Zhu, ZJH, Na Li, Hai‐Jian Sun, and Qing‐Bo Lu designed the experiment plans, wrote and reviewed the manuscript. Xue‐Xue Zhu, Xin‐Yu Meng, Fang‐Ming Wang, Xiao‐Ying Chai, Guo Chen, An‐Jing Xu, Jia‐Bao Su, and Hong‐Bo Qiu carried out the experiments and data analyses. Qing‐Yi Sun, Zhi‐Jun Han, Yao Wang, Zhuo‐Lin Lv, Yao Liu, and Yuan Zhang provided technical support and intelligence. Hai‐Jian Sun and Qing‐Bo Lu served as the lead contact for this work.

## CONFLICT OF INTEREST STATEMENT

The authors declare no competing interests.

## ETHICS STATEMENT

All experiments in mice were reviewed and approved by Animal Care and Use Committee of the China Pharmaceutical University (202101017).

## Supporting information



Supporting Information

## Data Availability

Source data for all main figures and extended data figures are supplied with this paper. Experimental data supporting the plots within this paper and other findings of this study are available from the corresponding author upon reasonable request.

## References

[1] Sun HJ , Wu ZY , Nie XW , Bian JS . Role of endothelial dysfunction in cardiovascular diseases: the link between inflammation and hydrogen sulfide. Front Pharmacol. 2019;10:1568. 32038245 10.3389/fphar.2019.01568 PMC6985156

[2] Sun HJ , Ni ZR , Liu Y , et al. Deficiency of neutral cholesterol ester hydrolase 1 (NCEH1) impairs endothelial function in diet‐induced diabetic mice. Cardiovasc Diabetol. 2024;23:138. 38664801 10.1186/s12933-024-02239-6 PMC11046792

[3] Vergès B . Cardiovascular disease in type 1 diabetes, an underestimated danger: epidemiological and pathophysiological data. Atherosclerosis. 2024;394:117158. 37369617 10.1016/j.atherosclerosis.2023.06.005

[4] Naderi‐Meshkin H , Setyaningsih WAW . Endothelial cell dysfunction: onset, progression, and consequences. Front Biosci (Landmark edition). 2024;29:223. 10.31083/j.fbl2906223 38940049

[5] Wang ZC , Niu KM , Wu YJ , Du KR , Qi LW . A dual Keap1 and p47(phox) inhibitor Ginsenoside Rb1 ameliorates high glucose/ox‐LDL‐induced endothelial cell injury and atherosclerosis. Cell Death Dis. 2022;13:824. 36163178 10.1038/s41419-022-05274-x PMC9512801

[6] Zhang JR , Sun HJ . Roles of circular RNAs in diabetic complications: from molecular mechanisms to therapeutic potential. Gene. 2020;763:145066. 32827686 10.1016/j.gene.2020.145066

[7] Sun HJ , Xiong SP . Hydrogen sulfide in diabetic complications revisited: the state of the art, challenges, and future directions. Antioxid Redox Signal. 2023;38:18‐44. 36310428 10.1089/ars.2022.0028

[8] Sun HJ , Wu ZY , Nie XW , Wang XY , Bian JS . An updated insight into molecular mechanism of hydrogen sulfide in cardiomyopathy and myocardial ischemia/reperfusion injury under diabetes. Front Pharmacol. 2021;12:651884. 34764865 10.3389/fphar.2021.651884 PMC8576408

[9] Sun HJ , Hou B , Wang X , Zhu XX , Li KX , Qiu LY . Endothelial dysfunction and cardiometabolic diseases: role of long non‐coding RNAs. Life Sci. 2016;167:6‐11. 27838210 10.1016/j.lfs.2016.11.005

[10] Miao G , Zhao H , Guo K , Cheng J , Zhang S , Zhang X , Cai Z , Miao H , Shang Y . Mechanisms underlying attenuation of apoptosis of cortical neurons in the hypoxic brain by flavonoids from the stems and leaves of Scutellaria baicalensis Georgi. Neural Regen Res. 2014;9:1592‐1598. 25368645 10.4103/1673-5374.141784 PMC4211200

[11] Zhang X , Lee W , Bian JS . Recent advances in the study of Na(+)/K(+)‐ATPase in neurodegenerative diseases. Cells. 2022:11;4075. 10.3390/cells11244075 PMC9777075 36552839

[12] Huang S , Dong W , Lin X , Bian J . Na+/K+‐ATPase: ion pump, signal transducer, or cytoprotective protein, and novel biological functions. Neural Regen Res. 2024;19:2684‐2697. 38595287 10.4103/NRR.NRR-D-23-01175 PMC11168508

[13] Sun HJ , Tan JX , Shan XD , et al. DR region of NKAα1 is a target to ameliorate hepatic lipid metabolism disturbance in obese mice. Metabolism. 2023;145:155579. 37127227 10.1016/j.metabol.2023.155579

[14] Leng B , Deng L , Tan J , et al. Targeting the Na(+)/K(+) ATPase DR‐region with DR‐Ab improves doxorubicin‐induced cardiotoxicity. Free Radical Biol Med. 2023;204:38‐53. 37100355 10.1016/j.freeradbiomed.2023.04.008

[15] Huang S , Dong W , Lin X , et al. Disruption of the Na(+)/K(+)‐ATPase‐purinergic P2×7 receptor complex in microglia promotes stress‐induced anxiety. Immunity. 2024;57:495‐512. e11. 38395698 10.1016/j.immuni.2024.01.018

[16] Zhang X , Li G , Chen H , Nie XW , Bian JS . Targeting NKAα1 to treat Parkinson's disease through inhibition of mitophagy‐dependent ferroptosis. Free Radical Biol Med. 2024;218:190‐204. 38574977 10.1016/j.freeradbiomed.2024.04.002

[17] Zhu M , Cao L , Xiong S , Sun H , Wu Z , Bian JS . Na(+)/K(+)‐ATPase‐dependent autophagy protects brain against ischemic injury. Signal Transduct Target Ther. 2020;5:55. 32433549 10.1038/s41392-020-0153-7 PMC7237650

[18] Pulgar VM , Jeffers AB , Rashad HM , Diz DI , Aileru AA . Increased constrictor tone induced by ouabain treatment in rats. J Cardiovasc Pharmacol. 2013;62:174‐183. 23615157 10.1097/FJC.0b013e3182955d33 PMC3893306

[19] Cui X , Liu X , Feng H , Zhao S , Gao H . Grape seed proanthocyanidin extracts enhance endothelial nitric oxide synthase expression through 5'‐AMP activated protein kinase/Surtuin 1‐Krüpple like factor 2 pathway and modulate blood pressure in ouabain induced hypertensive rats. Biol Pharm Bull. 2012;35:2192‐2197. 22987017 10.1248/bpb.b12-00598

[20] Pagán RM , Prieto D , Hernández M , et al. Regulation of NO‐dependent acetylcholine relaxation by K+ channels and the Na+‐K+ ATPase pump in porcine internal mammary artery. Eur J Pharmacol. 2010;641:61‐66. 20519140 10.1016/j.ejphar.2010.05.004

[21] Bondarenko A , Sagach V . Na+‐K+‐ATPase is involved in the sustained ACh‐induced hyperpolarization of endothelial cells from rat aorta. Br J Pharmacol. 2006;149:958‐965. 17001300 10.1038/sj.bjp.0706913 PMC2014692

[22] Fiorim J , Ribeiro RF Jr , Azevedo BF , et al. Activation of K+ channels and Na+/K+ ATPase prevents aortic endothelial dysfunction in 7‐day lead‐treated rats. Toxicol Appl Pharmacol. 2012;262:22‐31. 22546088 10.1016/j.taap.2012.04.015

[23] Liu Q , Liu CQ , Yi WZ , et al. Ferroptosis contributes to microvascular dysfunction in diabetic retinopathy. Am J Pathol. 2024;194:1078‐1089. 38417697 10.1016/j.ajpath.2024.01.019

[24] Luo EF , Li HX , Qin YH , et al. Role of ferroptosis in the process of diabetes‐induced endothelial dysfunction. World J Diab. 2021;12:124‐137. 10.4239/wjd.v12.i2.124 PMC7839168 33594332

[25] Yan B , Belke D , Gui Y , Chen YX , Jiang ZS , Zheng XL . Pharmacological inhibition of MALT1 (mucosa‐associated lymphoid tissue lymphoma translocation protein 1) induces ferroptosis in vascular smooth muscle cells. Cell Death Discov. 2023;9:456. 38097554 10.1038/s41420-023-01748-9 PMC10721807

[26] James PF , Grupp IL , Grupp G , et al. Identification of a specific role for the Na,K‐ATPase alpha 2 isoform as a regulator of calcium in the heart. Mol Cell. 1999;3:555‐563. 10360172 10.1016/s1097-2765(00)80349-4

[27] Sun HJ , Cao L , Zhu MY , et al. DR‐region of Na(+)/K(+)‐ATPase is a target to ameliorate hepatic insulin resistance in obese diabetic mice. Theranostics. 2020;10:6149‐6166. 32483445 10.7150/thno.46053 PMC7255017

[28] Tian XY , Wong WT , Xu A , et al. Uncoupling protein‐2 protects endothelial function in diet‐induced obese mice. Circ Res. 2012;110:1211‐1216. 22461387 10.1161/CIRCRESAHA.111.262170

[29] Li Z , Li S , Hu L , Li F , et al. Mechanisms underlying action of xinmailong injection, a traditional Chinese medicine in cardiac function improvement. Afr J Trad, Complemen, Alternat Med. 2017;14:241‐252. 10.21010/ajtcam.v14i2.26 PMC5446449 28573241

[30] Magid R , Martinson D , Hwang J , Jo H , Galis ZS . Optimization of isolation and functional characterization of primary murine aortic endothelial cells. Endothelium. 2003;10:103‐109. 12791518 10.1080/10623320303364

[31] Luo JY , Cheng CK , He L , Pu Y , Zhang Y , Lin X . Endothelial UCP2 is a mechanosensitive suppressor of atherosclerosis. Circ Res. 2022;131:424‐441. 35899624 10.1161/CIRCRESAHA.122.321187 PMC9390236

[32] Zhu XX , Meng XY , Chen G , et al. Nesfatin‐1 enhances vascular smooth muscle calcification through facilitating BMP‐2 osteogenic signaling. Cell Commun Signal. 2024;22:488. 39394127 10.1186/s12964-024-01873-7 PMC11468037

[33] Ren XS , Tong Y , Qiu Y , et al. MiR155‐5p in adventitial fibroblasts‐derived extracellular vesicles inhibits vascular smooth muscle cell proliferation via suppressing angiotensin‐converting enzyme expression. J Extracell Vesicles. 2020;9:1698795. 31839907 10.1080/20013078.2019.1698795 PMC6896498

[34] Sun HJ , Ren XS , Xiong XQ , et al. NLRP3 inflammasome activation contributes to VSMC phenotypic transformation and proliferation in hypertension. Cell Death Dis. 2017;8:e3074. 28981106 10.1038/cddis.2017.470 PMC5680591

[35] Symons JD , McMillin SL , Riehle C , et al. Contribution of insulin and Akt1 signaling to endothelial nitric oxide synthase in the regulation of endothelial function and blood pressure. Circ Res. 2009;104:1085‐1094. 19342603 10.1161/CIRCRESAHA.108.189316 PMC2936913

[36] Sun HJ , Chen D , Wang PY , et al. Salusin‐β is involved in diabetes mellitus‐induced endothelial dysfunction via degradation of peroxisome proliferator‐activated receptor gamma. Oxid Med Cell Longev. 2017;2017:6905217. 29359008 10.1155/2017/6905217 PMC5735326

[37] Zhu Q , Han Y , He Y , et al. Quercetin inhibits neuronal Ferroptosis and promotes immune response by targeting lipid metabolism‐related gene PTGS2 to alleviate breast cancer‐related depression. Phytomedicine. 2024;130:155560. 38815404 10.1016/j.phymed.2024.155560

[38] Zhang K , Wu Y , Chen G , Wang H , Liu Y , Zhou Y . Heat shock protein 27 deficiency promotes ferrous ion absorption and enhances acyl‐Coenzyme A synthetase long‐chain family member 4 stability to promote glioblastoma cell ferroptosis. Cancer Cell Int. 2023;23:5. 36639654 10.1186/s12935-023-02848-3 PMC9840324

[39] Huang Q , Ru Y . Identification of a targeted ACSL4 inhibitor to treat ferroptosis‐related diseases. Sci Adv. 2024;10:eadk1200. 38552012 10.1126/sciadv.adk1200 PMC10980261

[40] Lu QB , Du Q , Wang HP , Tang ZH , Wang YB , Sun HJ . Salusin‐β mediates tubular cell apoptosis in acute kidney injury: involvement of the PKC/ROS signaling pathway. Redox Biol. 2020;30:101411. 31884071 10.1016/j.redox.2019.101411 PMC6939056

[41] Shangguan J , Liu G , Xiao L , Zhang W , Zhu X , Li L . Meteorin‑like/meteorin‑β protects against cardiac dysfunction after myocardial infarction in mice by inhibiting autophagy. Exp Therap Med. 2024;28:293. 38827476 10.3892/etm.2024.12582 PMC11140287

[42] Xue L , Xu F , Meng L , et al. Acetylation‐dependent regulation of mitochondrial ALDH2 activation by SIRT3 mediates acute ethanol‐induced eNOS activation. FEBS Lett. 2012;586:137‐142. 22155639 10.1016/j.febslet.2011.11.031

[43] Zhu W , Yang B , Fu H , et al. Flavone inhibits nitric oxide synthase (NOS) activity, nitric oxide production and protein S‐nitrosylation in breast cancer cells. Biochem Biophys Res Commun. 2015;458:590‐595. 25680459 10.1016/j.bbrc.2015.01.154

[44] Bao MH , Li JM , Luo HQ , et al. NF‐κB‐regulated miR‐99a modulates endothelial cell inflammation. Mediators Inflamm. 2016;2016:5308170. 27403035 10.1155/2016/5308170 PMC4923609

[45] Sun H , Zhu X , Zhou Y , Cai W , Qiu L . C1q/TNF‐related protein‐9 ameliorates Ox‐LDL‐induced endothelial dysfunction via PGC‐1α/AMPK‐mediated antioxidant enzyme induction. Int J Mol Sci. 2017;18. 10.3390/ijms18061097 PMC5485929 28587104

[46] Jung S , Myagmarjav D , Jo T , et al. Inhibitory role of TRIP‐Br1 oncoprotein in anticancer drug‐mediated programmed cell death via mitophagy activation. Int J Biol Sci. 2022;18:3859‐3873. 35813469 10.7150/ijbs.72138 PMC9254482

[47] Dong L , Xie J , Wang Y , et al. Mannose ameliorates experimental colitis by protecting intestinal barrier integrity. Nat Commun. 2022;13:4804. 35974017 10.1038/s41467-022-32505-8 PMC9381535

[48] Zhu XX , Fu X , Meng XY , et al. Gut microbiome and metabolites mediate the benefits of caloric restriction in mice after acute kidney injury. Redox Biol. 2024;77:103373. 39357422 10.1016/j.redox.2024.103373 PMC11471245

[49] Cao L , Xiong S , Wu Z , Ding L . Anti‐Na(+)/K(+)‐ATPase immunotherapy ameliorates α‐synuclein pathology through activation of Na(+)/K(+)‐ATPase α1‐dependent autophagy. Sci Adv. 2021;7. 10.1126/sciadv.abc5062 PMC7840131 33571110

[50] Shen J , San W , Zheng Y , et al. Different types of cell death in diabetic endothelial dysfunction. Biomed Pharmacother. 2023;168:115802. 37918258 10.1016/j.biopha.2023.115802

[51] Doll S , Proneth B , Tyurina YY , et al. ACSL4 dictates ferroptosis sensitivity by shaping cellular lipid composition. Nat Chem Biol. 2017;13:91‐98. 27842070 10.1038/nchembio.2239 PMC5610546

[52] Yuan H , Li X , Zhang X , Kang R , Tang D . Identification of ACSL4 as a biomarker and contributor of ferroptosis. Biochem Biophys Res Commun. 2016;478:1338‐1343. 27565726 10.1016/j.bbrc.2016.08.124

[53] Pei Z , Liu Y , Liu S , et al. FUNDC1 insufficiency sensitizes high fat diet intake‐induced cardiac remodeling and contractile anomaly through ACSL4‐mediated ferroptosis. Metabolism. 2021;122:154840. 34331963 10.1016/j.metabol.2021.154840

