# Supplementary material for: Sodium pump subunit NKAα1 protects against diabetic endothelial dysfunction by inhibiting ferroptosis through the autophagy‐lysosome degradation of ACSL4
Source: Clin Transl Med. 2025 Feb 4;15(2):e70221. doi: 10.1002/ctm2.70221 (PMC11995423; doi:10.1002/ctm2.70221)
Supplement: Supplementary file 1 — Supporting Information [file CTM2-15-e70221-s001.docx]

**Supplementary data**

**
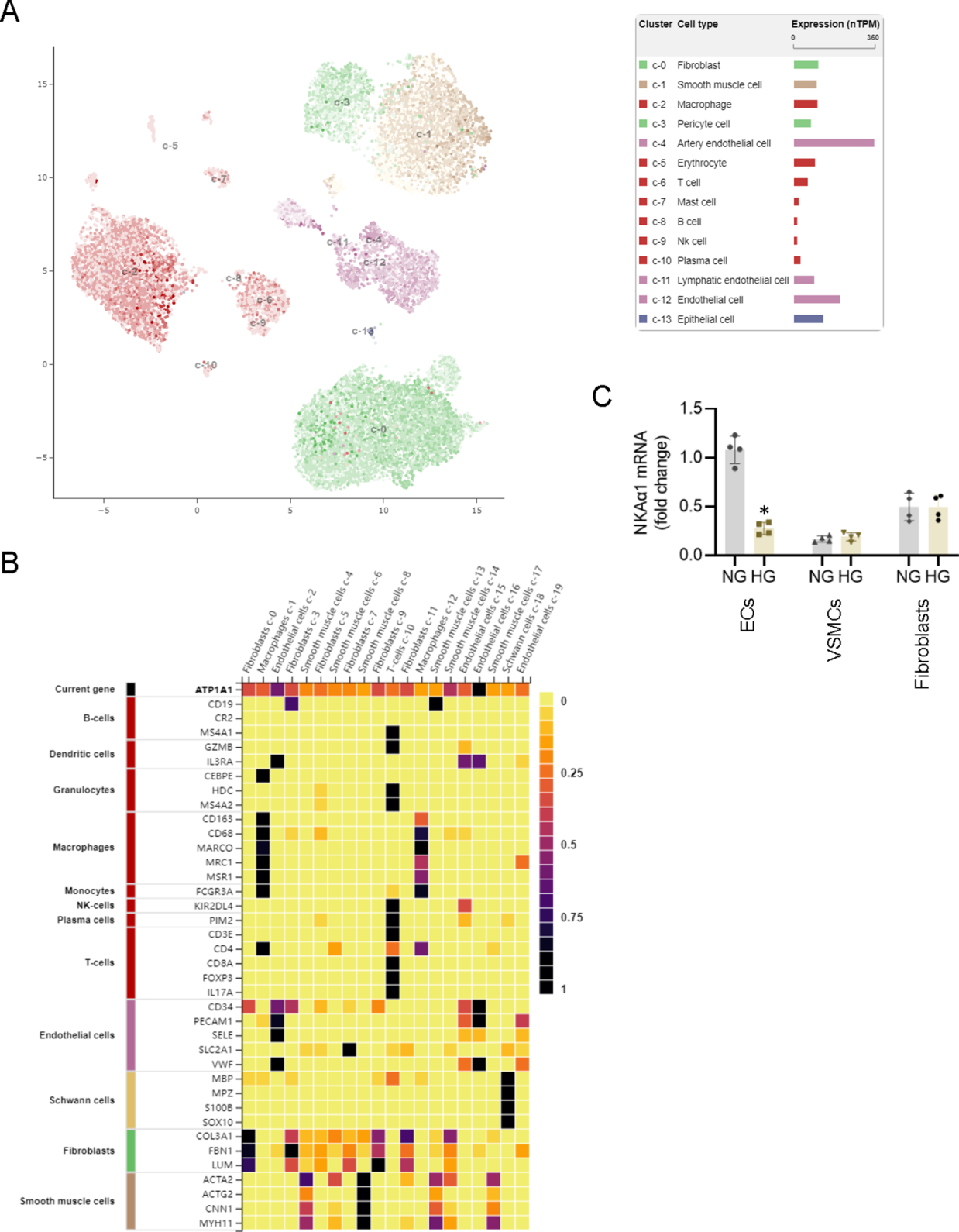
**

**Figure S1.** **The expression and distribution of NKAα1 in human aortae using the online database HUMAN PROTEIN ATLAS**. (**A,B**) The online database of HUMAN PROTEIN ATLAS shows the expression of PGK1 in different cell types in human aortae. (**C**) Effects of HG on the mRNA level of NKAα1 in primary ECs, vascular smooth muscle cells, and vascular fibroblasts. * P < 0.05 *versus* NG.

**
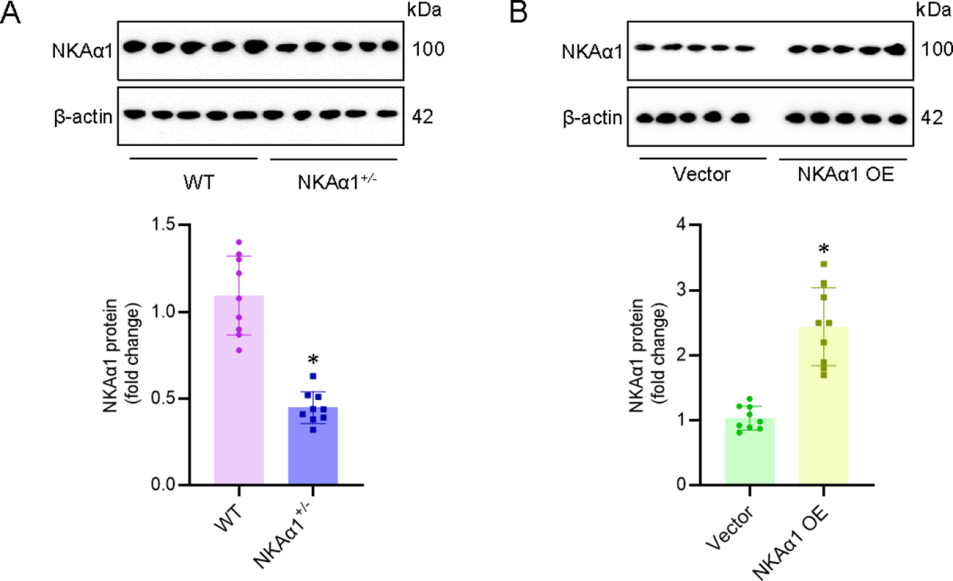
**

**Figure S2. Expression of NKAα1 in mouse aortae.** (**A**) Representative blot and quantitative analysis of NKAα1 in aortae from WT and NKAα1^+/-^ mice. (**B**) Representative blot and quantitative analysis of NKAα1 in aortae from control and NKAα1 overexpression mice. The P-value was calculated by unpaired two-tailed Student’s t-test. * P < 0.05 *versus* WT or Vector.


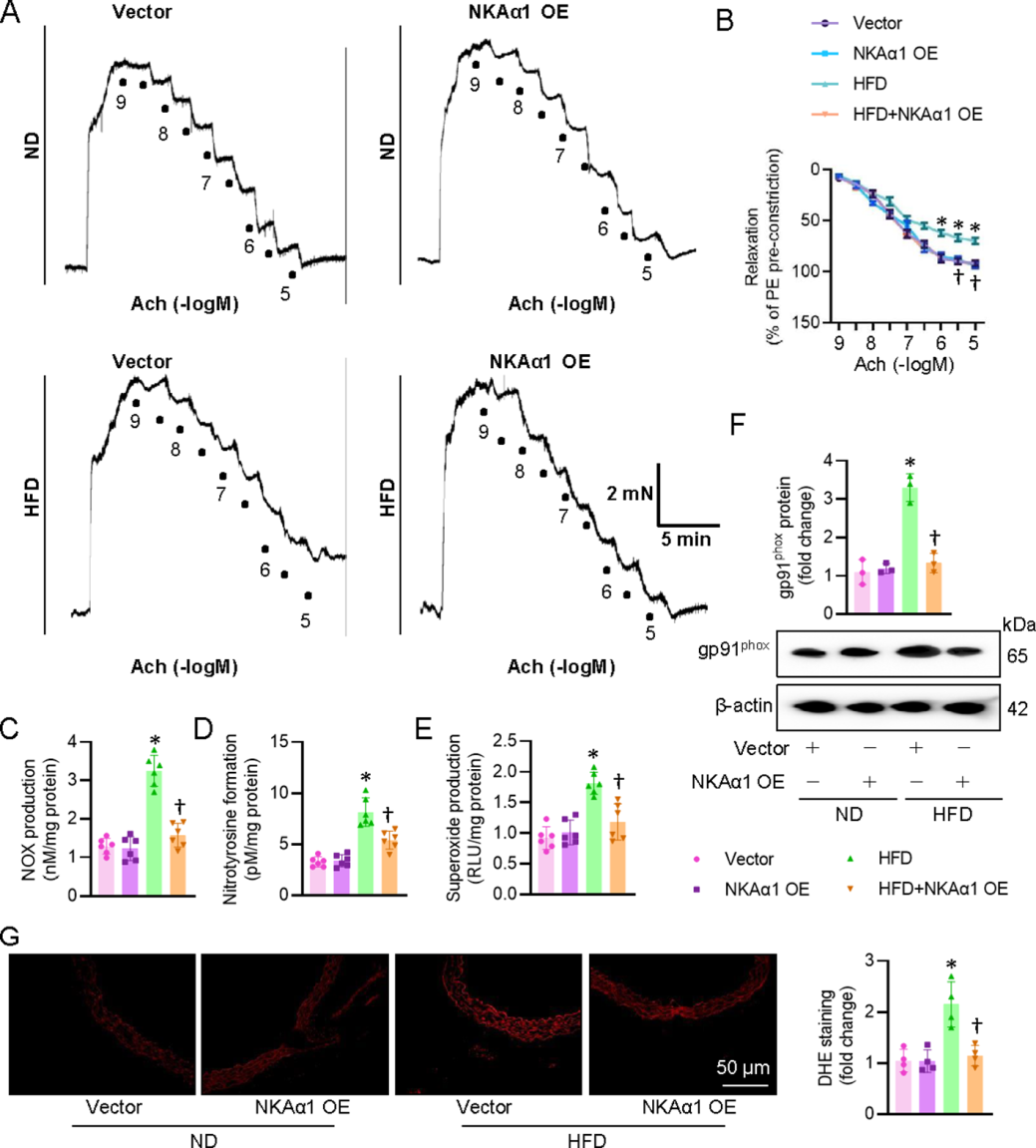


**Figure S3. Effects of NKAα1 overexpression on EDR and oxidative/nitrative stresses in aortic segments**. (**A, B**) Overexpression of NKAα1 improved EDR in diabetic mice. (**C**) NOX production. (**D**) Nitrotyrosine formation. (**E**) Superoxide production. (**F**) Representative blot and quantitative analysis of gp91^phox^ protein. (**G**) Representative image and quantitative analysis of DHE staining. Scale bar, 50 μm. Differences between groups were assessed with ANOVA followed by Bonferroni post-hoc test. *P < 0.05 *versus* Vector. †P < 0.05 *versus* HFD.

**
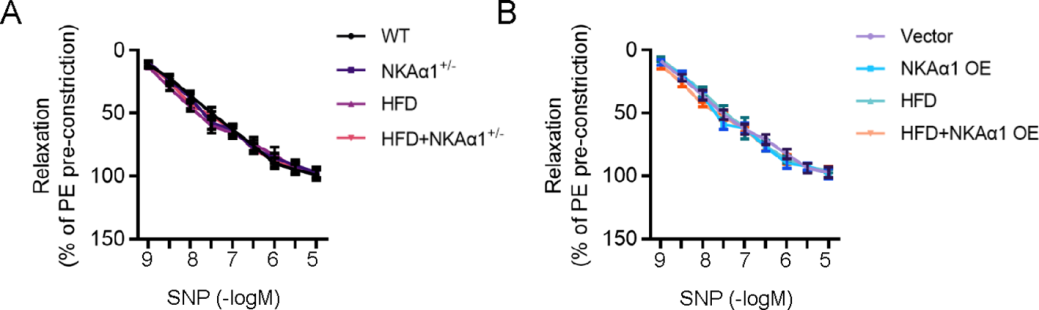
**

**Figure S4.** **NKAα1 loss (A) or overexpression (B) had minimal effects on endothelium-independent vasorelaxation**. n = 6. Differences between groups were assessed with ANOVA followed by Bonferroni post-hoc test.


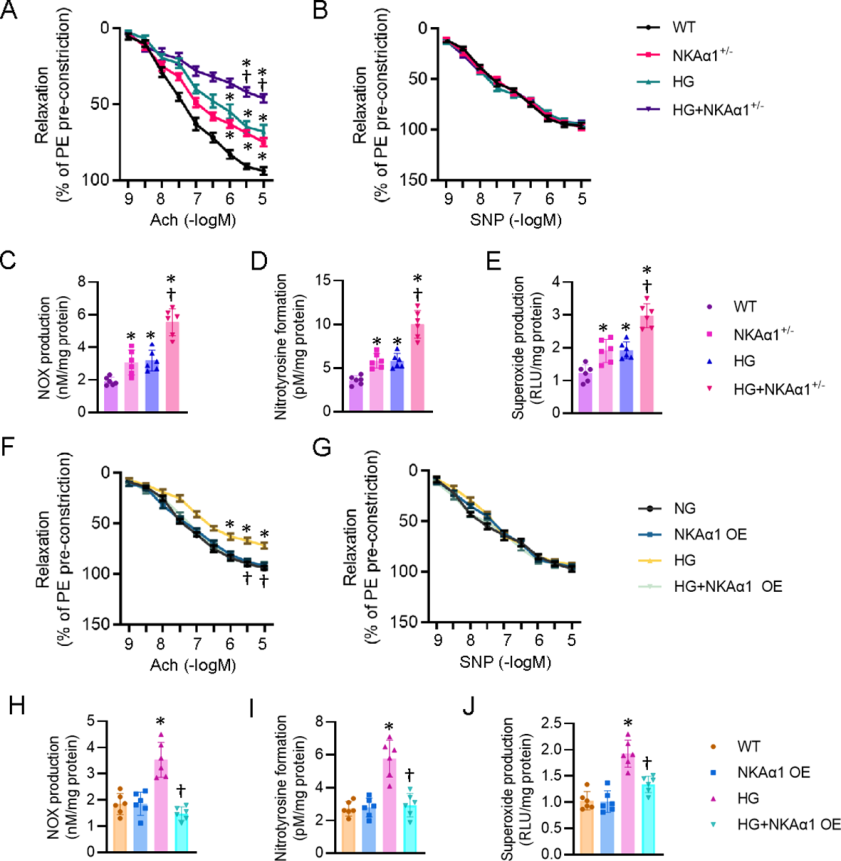


**Figure S5. Effects of NKAα1 on vascular relaxation in HG-incubated mouse aortae**. (**A**) EDR in aortic rings from NG (mannitol, 25 mM) or HG (D-glucose (25 mM)-induced mouse aortae with or without NKAα1. (**B**) Endothelium-independent vasorelaxation in aortic rings from NG (mannitol, 25 mM) or HG (D-glucose (25 mM)-induced mouse aortae with or without NKAα1. (**C**) NOX production. n = 6. (**D**) Nitrotyrosine formation. (**E**) Superoxide production. (**F**) EDR in aortic rings from NG- or HG-induced mouse aortae after overexpression of NKAα1. (**G**) Endothelium-independent relaxation in aortic rings from NG- or HG-induced mouse aortae after overexpression of NKAα1. (**H**) NOX production. (**I**) Nitrotyrosine formation. (**J**) Superoxide production. Differences between groups were assessed with ANOVA followed by Bonferroni post-hoc test. *P < 0.05 *versus* Wild-type (WT) or NG. †P < 0.05 *versus* HG.


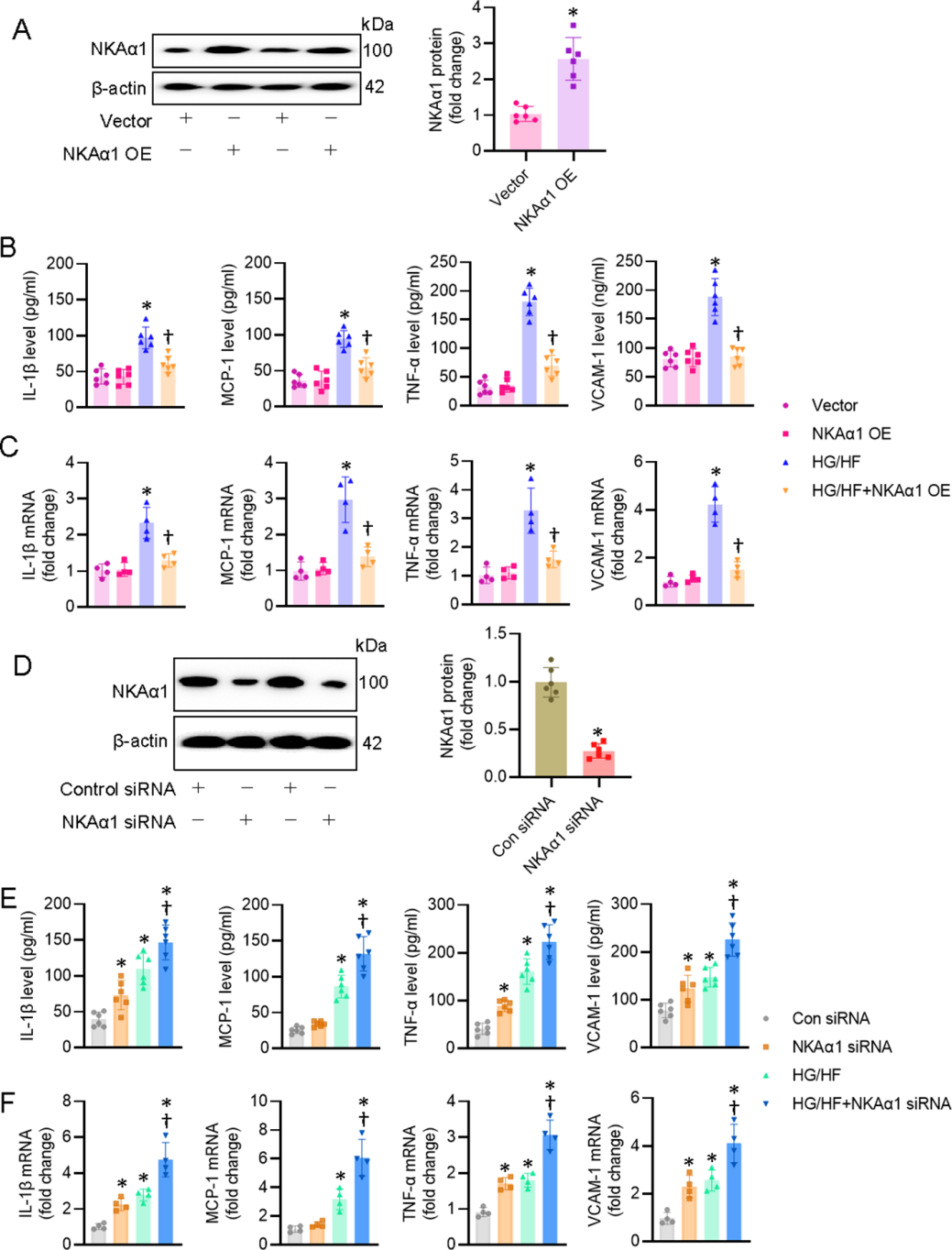


**Figure S6. Effects of NKAα1 overexpression and knockdown on inflammation response in ECs.** (**A**) Detection of NKAα1 overexpression efficiency. (**B**) The protein expression of IL-1β, MCP-1, TNF-α, and VCAM-1 measured by ELISA. (**C**) The mRNA levels of IL-1β, MCP-1, TNF-α, and VCAM-1. (**D**) Detection of NKAα1 knockdown efficiency. (**E**) The protein expression of IL-1β, MCP-1, TNF-α, and VCAM-1 measured by ELISA. (**F**) The mRNA levels of IL-1β, MCP-1, TNF-α, and VCAM-1. The P-value was calculated by unpaired two-tailed Student’s t-test (A, D). Differences between groups were assessed with ANOVA followed by Bonferroni post-hoc test (B-C, E-F). *P < 0.05 *versus* Con siRNA or Vector. †P < 0.05 *versus* HG/HF.

**
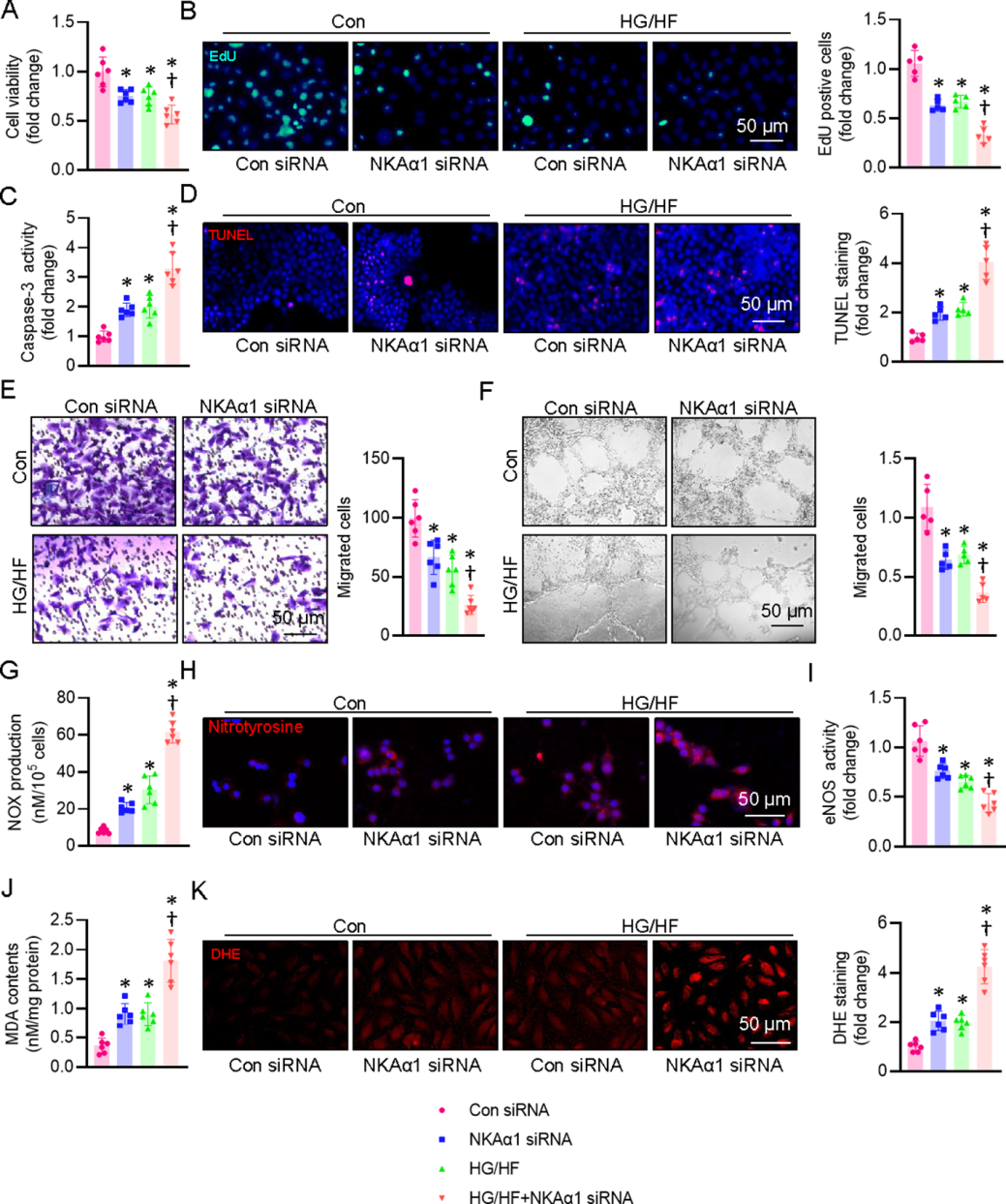
**

**Figure S7. Effects of NKAα1 knockdown on HG/HF-evoked EC injury *in vitro*.** (**A**) Cell viability assessed by CCK-8 assay. (**B**) Cell viability assessed EdU incorporation assay. Scale bar, 50 μm. (**C**) Caspase-3 activity. (**D**) Cell apoptosis assessed by TUNEL analysis. Scale bar, 50 μm. (**E**) The migration of HUVECs. Scale bar, 50 μm. (**F**) The angiogenesis of HUVECs. Scale bar, 50 μm. (**G**) NOX production. (**H**) Representative immunofluorescence staining of 3-Nitrotyrosine. Scale bar, 50 μm. (**I**) eNOS activity. (**J**) MDA contents. (**K**) Representative image and quantitative analysis of DHE staining. Scale bar, 50 μm. Differences between groups were assessed with ANOVA followed by Bonferroni post-hoc test. *P < 0.05 *versus* Con siRNA. †P < 0.05 *versus* HG/HF.

**
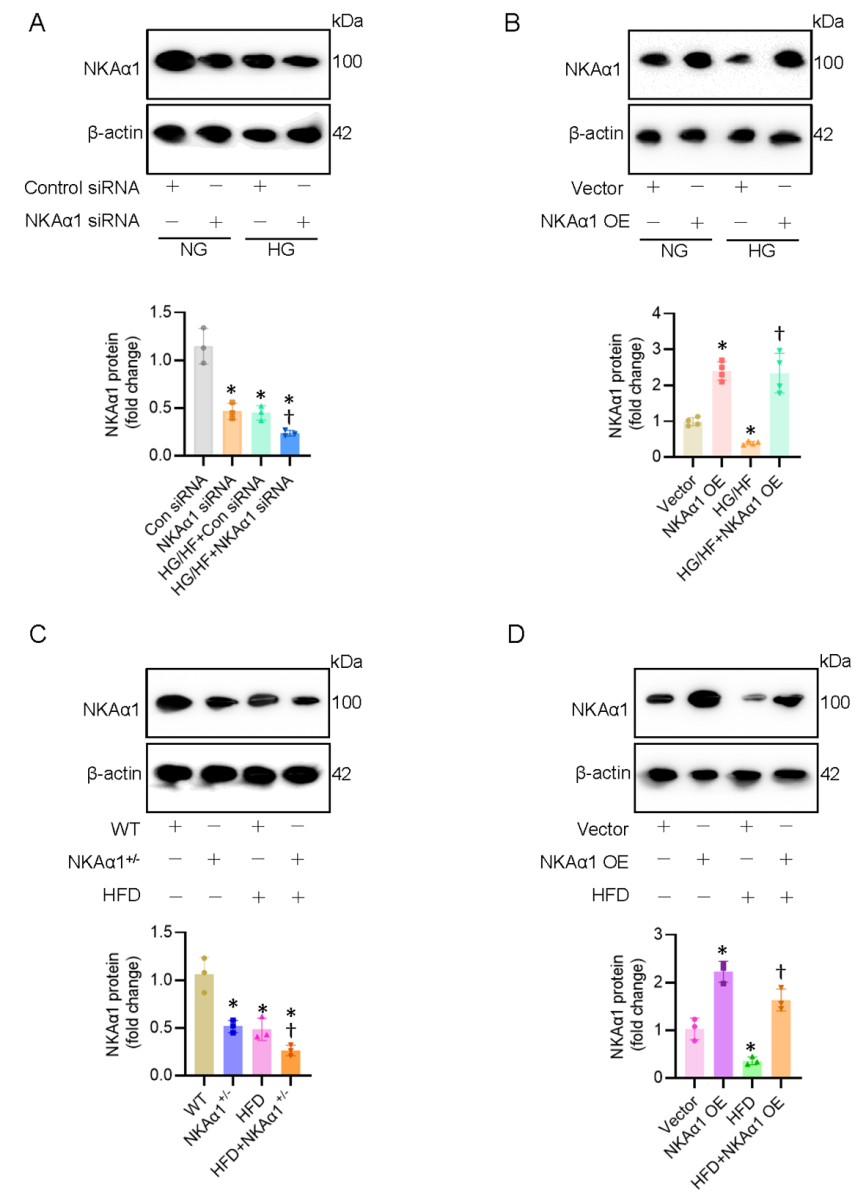
**

**Figure S8. Effects of NKAα1 knockdown and overexpression on the protein expression of NKAα1 in ECs and aortae.** (**A**) Detection of NKAα1 downregulation efficiency in ECs. (**B**) Detection of NKAα1 overexpression efficiency in ECs. (**C**) Detection of NKAα1 downregulation efficiency in aortae. (**D**) Detection of NKAα1 overexpression efficiency in aortae. *P < 0.05 *versus* Con siRNA, Vector, or WT. †P < 0.05 *versus* HG/HF+Con siRNA, HG/HF, or HFD.


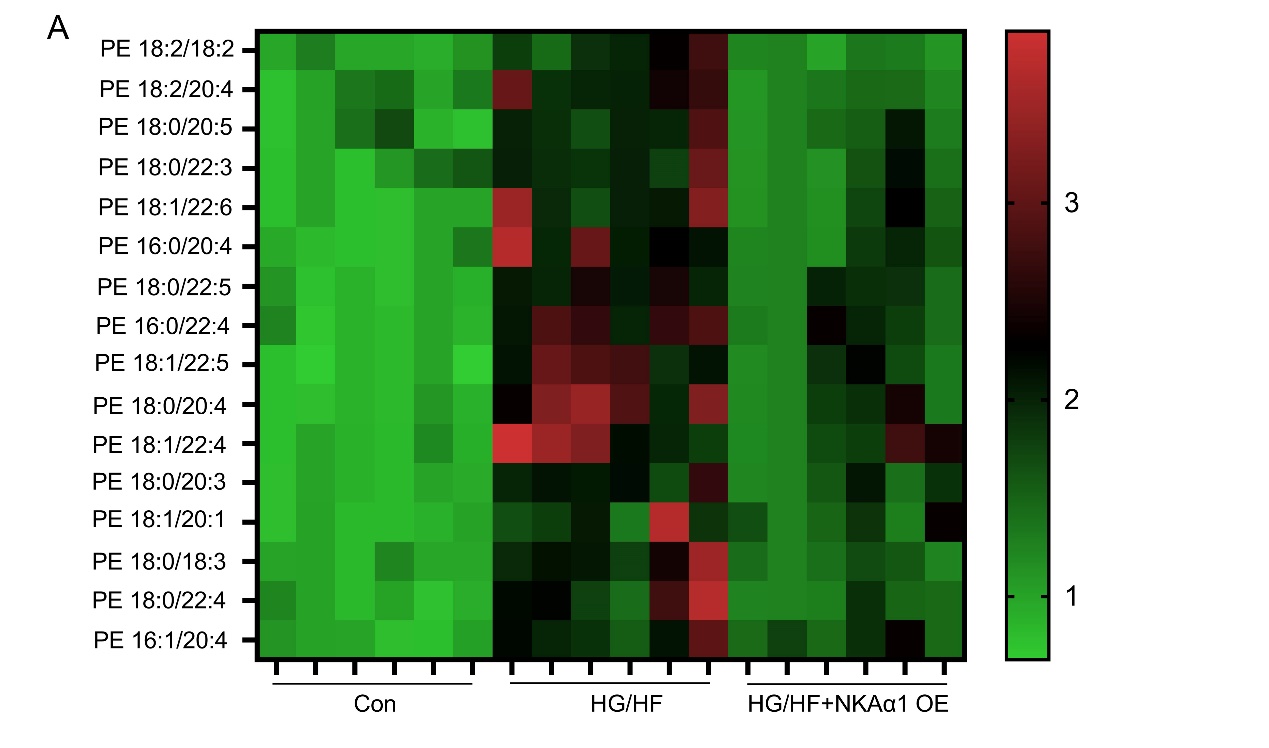


**Figure S9. Effects of NKAα1 overexpression on lipid metabolism in ECs.** (**A**) Heatmap of major PE species in HUVECs after NKAα1 overexpression. Each PE/PC species was normalized to the corresponding mean value. PE, phosphatidylethanolamine; PC, phosphatidyl cholines.


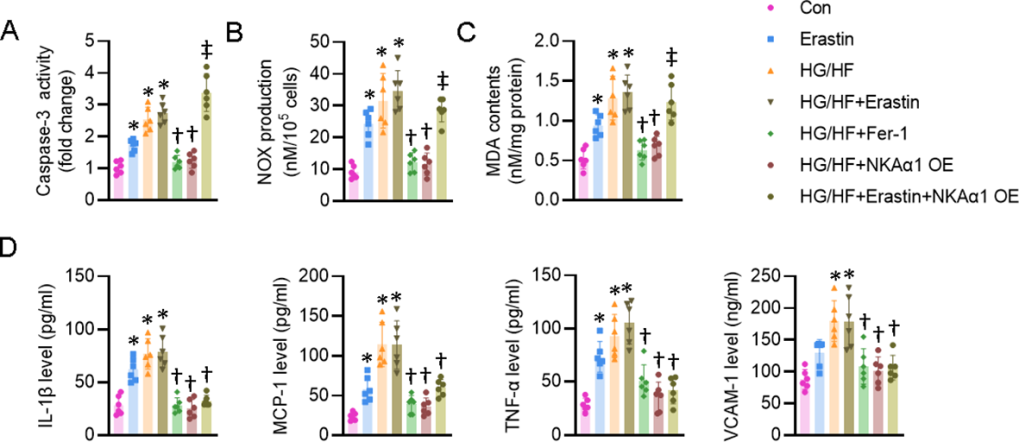


**Figure S10. Effects of erastin, a known ferroptosis inducer, on NKAα1 overexpression protection against endothelial injury induced by HG/HF**. (**A**) Caspase-3 activity. (**B**) NOX production. (**C**) MDA contents. (**D**) The protein expression of IL-1β, MCP-1, TNF-α, and VCAM-1 measured by ELISA. Differences between groups were assessed with ANOVA followed by Bonferroni post-hoc test. *P < 0.05 *versus* Con. †P < 0.05 *versus* HG/HF. ‡P < 0.05 *versus* HG/HF+NKAα1 OE (overexpression).


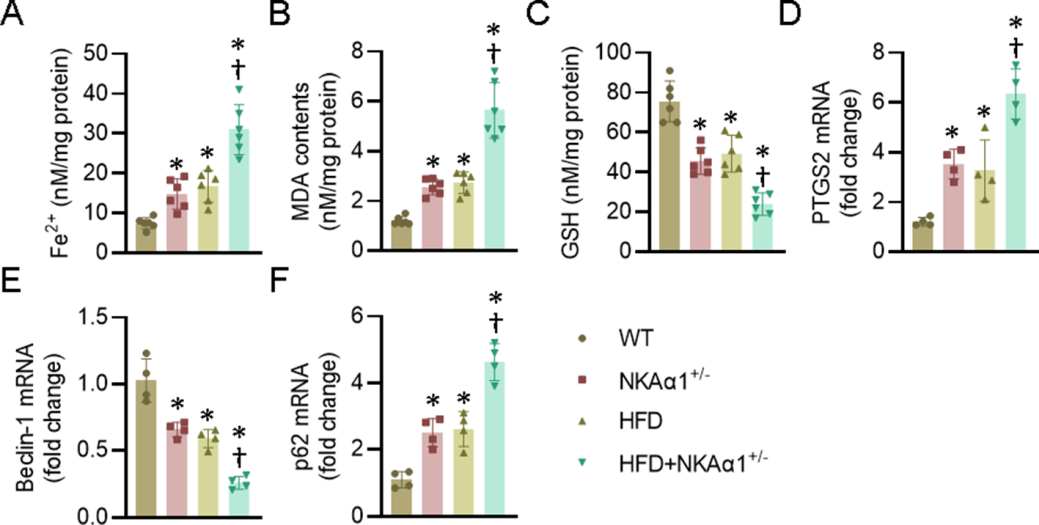


**Figure S11. Effects of NKAα1 knockdown on ferroptosis and autophagy in primary ECs.** (**A**) Fe^2+^ levels. (**B**) MDA contents. (**C**) GSH levels. (**D**) Relative mRNA level of PTGS2. (**E**) Relative mRNA level of Beclin-1. (**F**) Relative mRNA level of p62. *P < 0.05 *versus* WT. †P < 0.05 *versus* HFD.

**
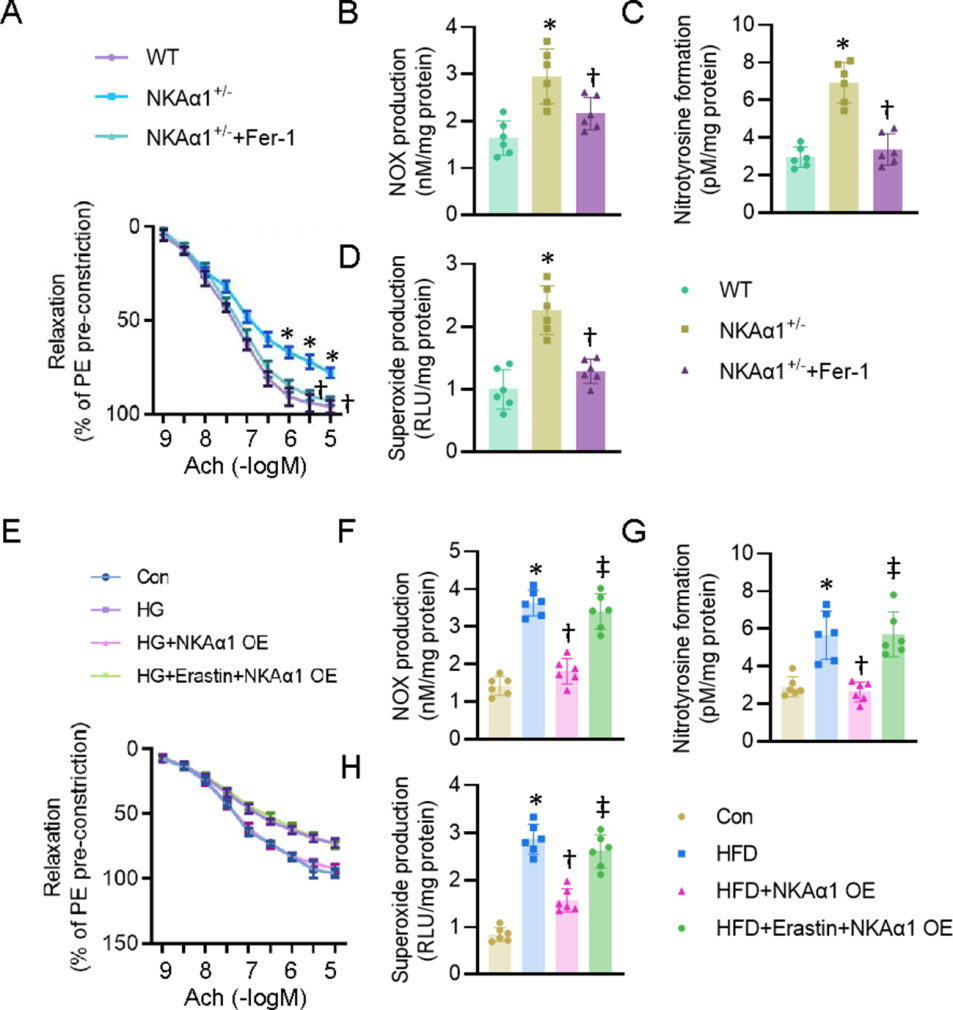
**

**Figure S12. Involvement of ferroptosis in NKAα1-mediated vascular function in mouse aortae.** (**A**) A ferroptosis inhibitor Fer-1 restored EDR in mouse aortae from *nkaα1*-deficient mice. (**B**) NOX production. (**C**) Nitrotyrosine formation. (**D**) Superoxide production. (**E**) Erastin, a known ferroptosis inducer, abolished the effects of NKAα1 overexpression on EDR in mouse aortae. (**F**) NOX production. (**G**) Nitrotyrosine formation. (**H**) Superoxide production. Differences between groups were assessed with ANOVA followed by Bonferroni post-hoc test. *P < 0.05 *versus* Con or WT. †P < 0.05 *versus* NKAα1***^+/-^*** or HFD. ‡P < 0.05 *versus* HFD+ NKAα1 OE (overexpression).

**
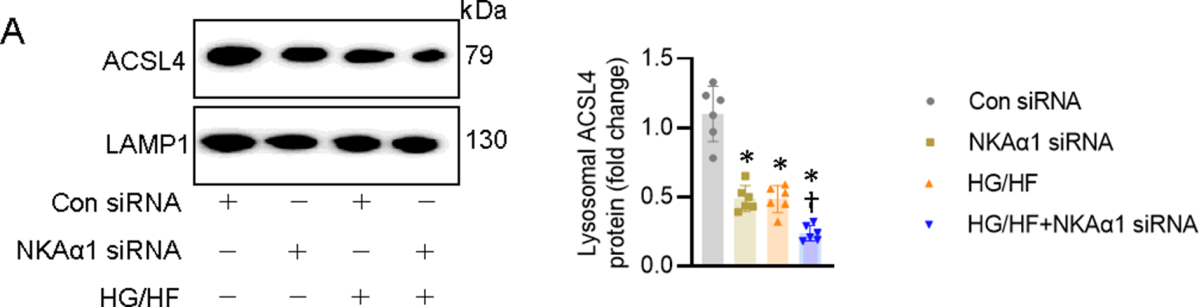
**

**Figure S13. Effects of NKAα1 knockdown on the abundance of ACSL4 in the lysosome of HUVECs.** n = 6. Differences between groups were assessed with ANOVA followed by Bonferroni post-hoc test. *P < 0.05 *versus* Con siRNA. †P < 0.05 *versus* HG/HF.


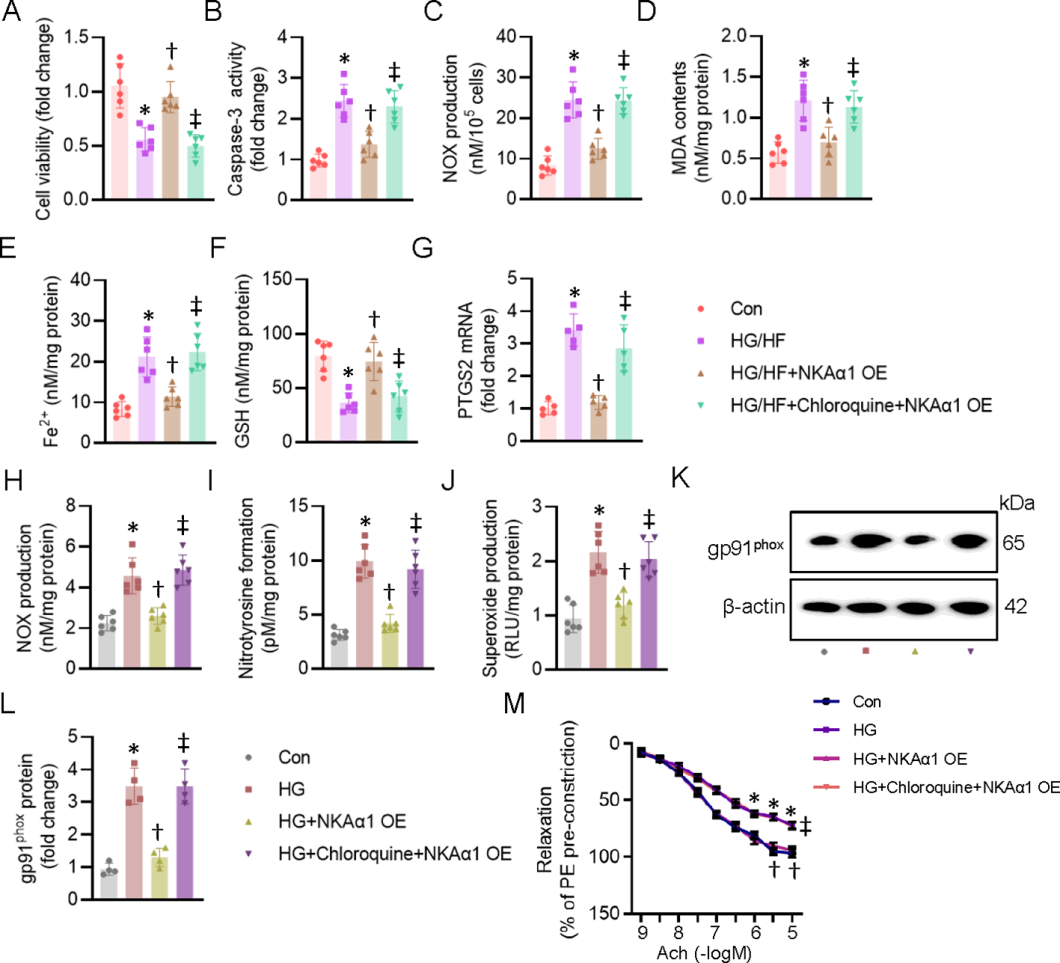


**Figure S14. Effects of chloroquine, a lysosome inhibitor, on NKAα1 overexpression protection against endothelial injury induced by HG/HF or HG**. (**A**) Cell viability. (**B**) Caspase-3 activity. (**C**) NOX production. (**D**) MDA contents. (**E**) Fe^2+^ contents. (**F**) GSH levels. (**G**) Relative mRNA level of PTGS2. (**H**) NOX production. (**I**) Nitrotyrosine formation. (**J**) Superoxide production. (**K, L**) Representative blot and quantitative analysis of gp91^phox^ protein. (**M**) EDR. Differences between groups were assessed with ANOVA followed by Bonferroni post-hoc test. *P < 0.05 *versus* Con. †P < 0.05 *versus* HG/HF. ‡P < 0.05 *versus* HG/HF+NKAα1 OE (overexpression).

**
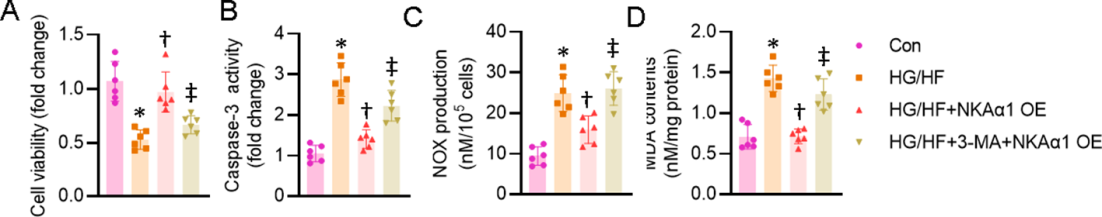
**

**Figure S15. Effects of 3-MA on NKAα1 overexpression protection against endothelial injury induced by HG/HF**. (**A**) Cell viability. (**B**) Caspase-3 activity. n = 6. (**C**) NOX production. (**D**) MDA contents. Differences between groups were assessed with ANOVA followed by Bonferroni post-hoc test. *P < 0.05 *versus* Con. †P < 0.05 *versus* HG/HF. ‡P < 0.05 *versus* HG/HF+NKAα1 OE (overexpression).


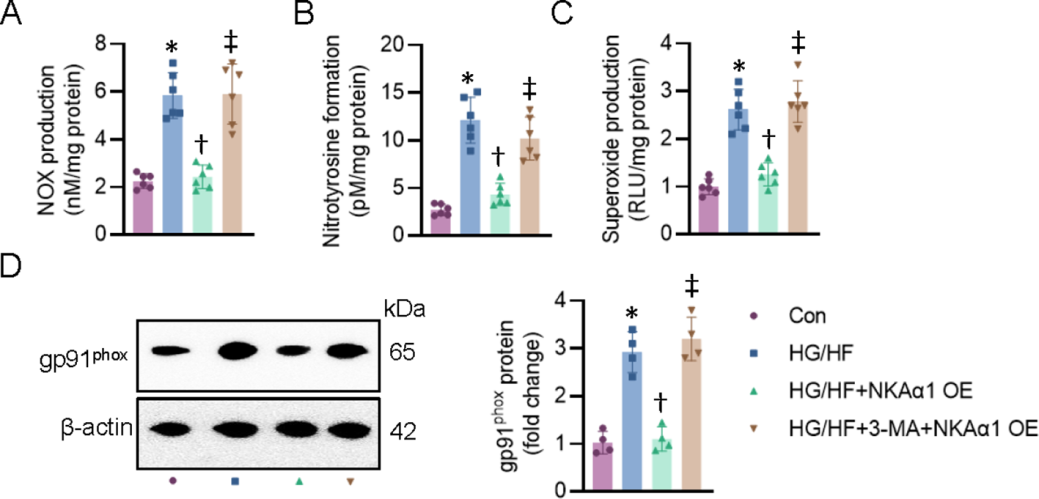


**Figure S16. Effects of 3-MA on NKAα1 overexpression protection against endothelial injury in mouse aortae induced by HG**. (**A**) NOX production. (**B**) Nitrotyrosine formation. (**C**) Superoxide production. (**D, E**) Representative blot and quantitative analysis of gp91^phox^ protein. Differences between groups were assessed with ANOVA followed by Bonferroni post-hoc test. *P < 0.05 *versus* Con. †P < 0.05 *versus* HG. ‡P < 0.05 *versus* HG +NKAα1 OE (overexpression).


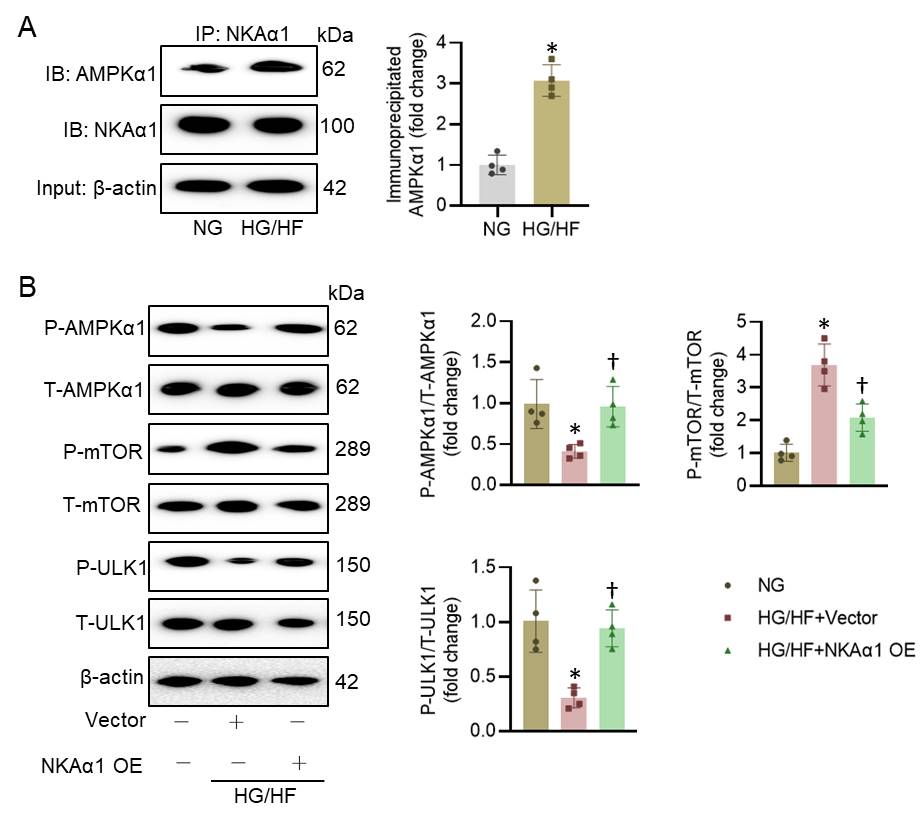


**Figure S17. Effects of NKAα1 overexpression on the AMPK/mTOR/ULK1 signaling pathway in ECs**. (**A**) HG/HF exposure increased the interaction of NKAα1 with AMPKα1. (**B**) Representative blot and quantitative analysis of P-AMPK, P-mTOR, and P-ULK1 in HG/HF-exposed ECs after NKAα1 overexpression. *P < 0.05 *versus* NG. †P < 0.05 *versus* HG/HF+Vector.

**
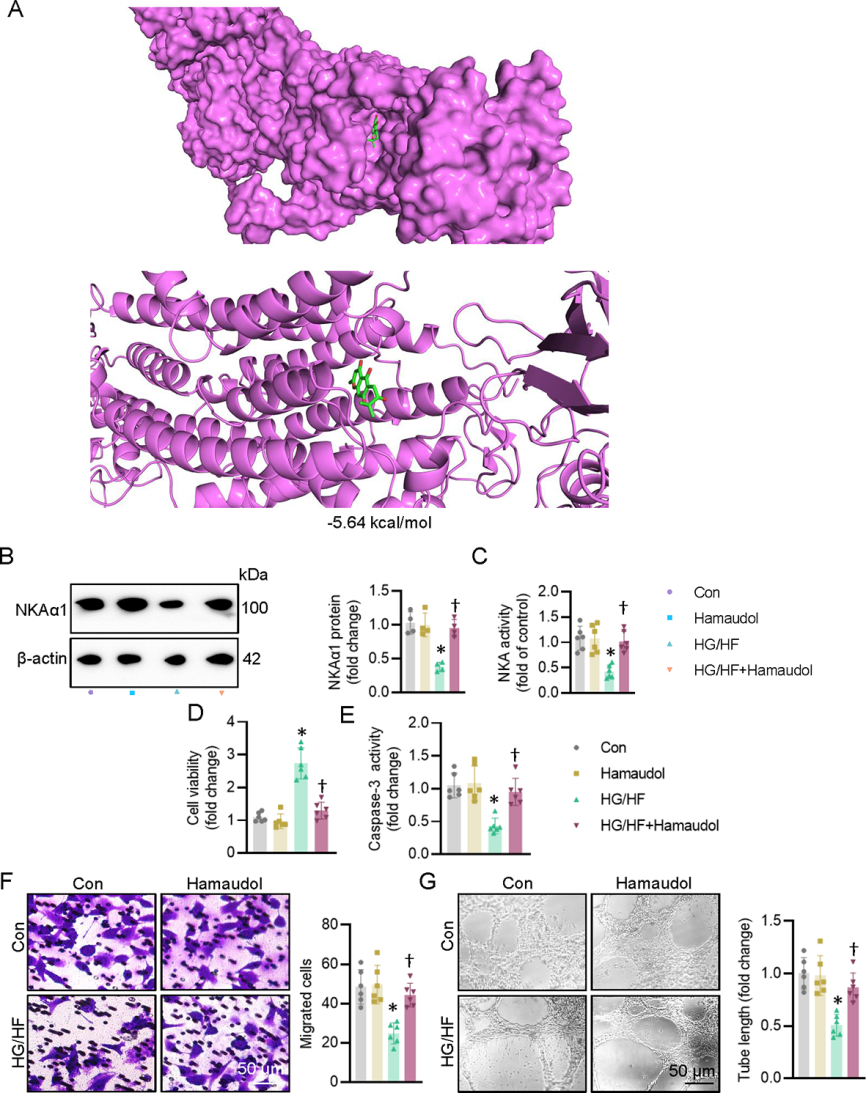
**

**Figure S18. Hamaudol attenuated HG/HF-induced injury in HUVECs.** (**A**) Molecular docking showing the direct binding of Hamaudol to NKAα1. (**B**) Hamaudol restored the protein expression of NKAα1. (**C**) Hamaudol restored the activity of NKA. (**D**) Cell viability assessed by CCK-8 assay. (**E**) Caspase-3 activity. (**F**) The migration of HUVECs. Scale bar, 50 μm. (**G**) The angiogenesis of HUVECs. Scale bar, 50 μm. Differences between groups were assessed with ANOVA followed by Bonferroni post-hoc test. *P < 0.05 *versus* Con. †P < 0.05 *versus* HG/HF.

**
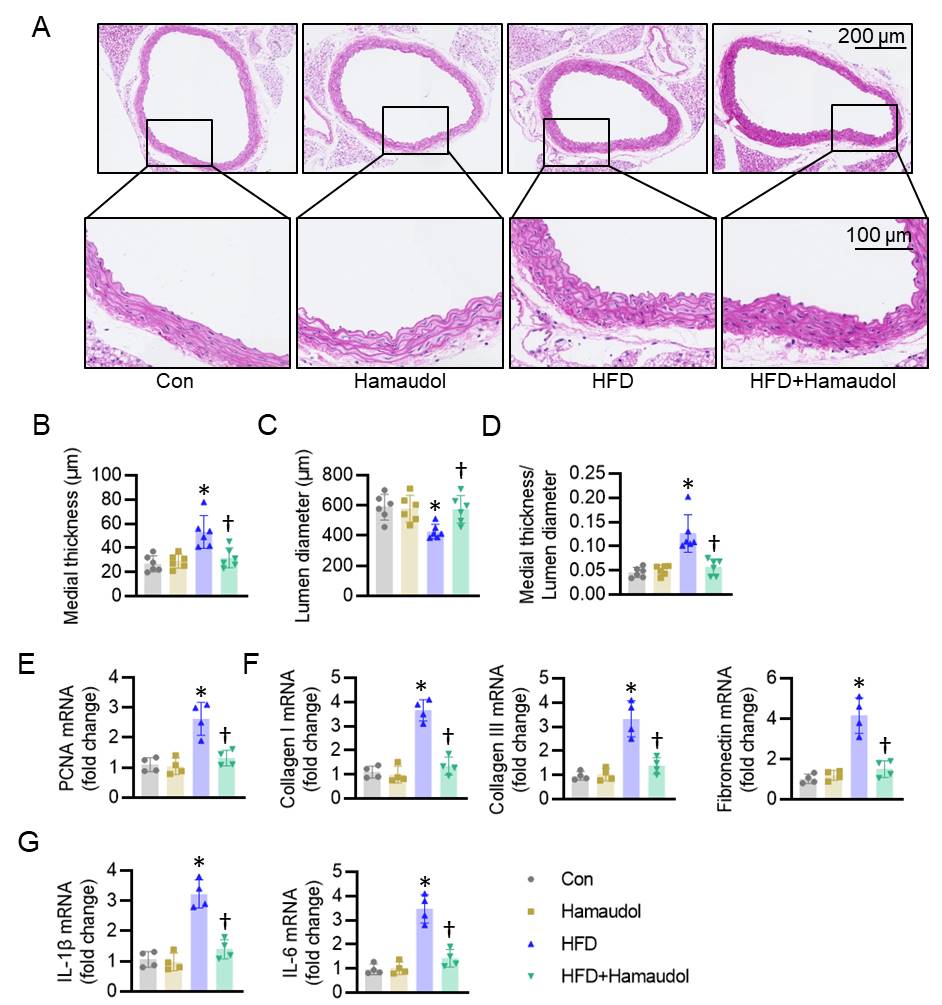
Figure S19. Hamaudol ameliorates vascular remodeling in HFD mice.** (**A**) Representative H&E staining of mouse aortae. (**B**) Medial thickness of mouse aortae. (**C**) Medial thickness of lumen diameter. (**D**) The medial thickness/lumen diameter ratio. (**E**) Relative mRNA level of PCNA. (**F**) Relative mRNA level of Collagen I, Collagen III, and Fibronectin. (**G**) Relative mRNA level of IL-1β and IL-6. *P < 0.05 *versus* Con. †P < 0.05 *versus* HFD.

**Table S1.** **Primer sequences for PCR analysis in HUVECs**

| Gene | Sequence |
| --- | --- |
| *NKAα1* | 5′-TCCATTATAATAGCCATCTT-3′ (Forward) |
|  | 5′-AATGAAGCATGTAGCTCTA-3′ (Reverse) |
| *NKAα2* | 5′-AGCTGAACTTTCCCACGGAG-3′ (Forward) |
|  | 5′-CTGATATGATGCCCACGCCT-3′ (Reverse) |
| *NKAα3* | 5′-TCAAGAAGGAGGTGGCTATG-3′(Forward) |
|  | 5′-GAGAAGCAGCCAGTGATGAT-3′ (Reverse) |
| *PTGS2* | 5'-CTGGCGCTCAGCCATACAG-3' (Forward) |
|  | 5'-CGCACTTATACTGGTCAAATCCC-3' (Reverse) |
| *Fth1* | 5'-ACATCAAGAAGGTGGTGAAGC-3' (Forward) |
|  | 5'-AAGGTGGAAGAGTGGGAGTTG-3' (Reverse) |
| *ACSL4* | 5'-CATCCCTGGAGCAGATACTCT-3' (Forward) |
|  | 5'-TCACTTAGGATTTCCCTGGTCC-3' (Reverse) |
| *GPX4* | 5' -TGGGTTGGCTGCTTGTG-3' (Forward) |
|  | 5' -GCGTGGGCAGGATGAAG-3' (Reverse) |
| *IL-1β* | 5' -ATGATGGCTTATTACAGTGGCAA-3' (Forward) |
|  | 5' -GTCGGAGATTCGTAGCTGGA-3' (Reverse) |
| *MCP-1* | 5' -CAGCCAGATGCAATCAATGCC-3' (Forward) |
|  | 5' -TGGAATCCTGAACCCACTTCT-3' (Reverse) |
| *TNF-α* | 5' -AAGCACACTGGTTTCCACACT-3' (Forward) |
|  | 5' -TGGGTCCCTGCATATCCGTT-3' (Reverse) |
| *VCAM-1* | 5' -GGGAAGATGGTCGTGATCCTT-3' (Forward) |
|  | 5' -TCTGGGGTGGTCTCGATTTTA-3' (Reverse) |
| *GAPDH* | 5'-CACTGCCACCCAGAAGA-3' (Forward) |
|  | 5'-GCTTCCCGTTCAGCTCA-3' (Reverse) |

**Table S2.** **Primer sequences for PCR analysis in mice**.

| Gene | Sequence |
| --- | --- |
| *NKAα1* | 5′-GATCAGCATGGCCTATGGACAG-3′ (Forward) |
|  | 5′-ACCGTTCTCAGCCAGAATCACA-3′ (Reverse) |
| *NKAα2* | 5′-GGCCGAAAATACCAAGTGGAT-3′ (Forward) |
|  | 5′-TGTCCTGAGCTCGCTGATTG-3′ (Reverse) |
| *NKAα3* | 5′-TCAAGAAGGAGGTGGCTATG-3′(Forward) |
|  | 5′-GAGAAGCAGCCAGTGATGAT-3′(Reverse) |
| *PTGS2* | 5'-TGCACTATGGTTACAAAAGCTGG-3' (Forward) |
|  | 5'-TCAGGAAGCTCCTTATTTCCCTT-3' (Reverse) |
| *Fth1* | 5'-CAAGTGCGCCAGAACTACCA-3' (Forward) |
|  | 5'-ACAGATAGACGTAGGAGGCATAC-3' (Reverse) |
| *ACSL4* | 5'-CCTGAGGGGCTTGAAATTCAC-3' (Forward) |
|  | 5'-GTTGGTCTACTTGGAGGAACG-3' (Reverse) |
| *GPX4* | 5'-TGTGCATCCCGCGATGATT-3' (Forward) |
|  | 5'-CCCTGTACTTATCCAGGCAGA-3' (Reverse) |
| *Atg12* | 5'-TCCCCGGAACGAGGAACTC-3' (Forward) |
|  | 5'-TTCGCTCCACAGCCCATTTC-3' (Reverse) |
| *Atg13* | 5'-CTTCTCGCTATTACAAGGGTGAC-3’ (Forward) |
|  | 5'-CCATTCAGTTGAACTTCCCCAAA-3’ (Reverse) |
| *Beclin-1* | 5'-ATGGAGGGGTCTAAGGCGTC-3’ (Forward) |
|  | 5'-TGGGCTGTGGTAAGTAATGGA-3’ (Reverse) |
| *ULK1* | 5'-AAGTTCGAGTTCTCTCGCAAG-3’ (Forward) |
|  | 5'-ACCTCCAGGTCGTGCTTCT-3’ (Reverse) |
| *p62* | 5'-GAGGCACCCCGAAACATGG-3’ (Forward) |
|  | 5'-ACTTATAGCGAGTTCCCACCA-3’ (Reverse) |
| *GAPDH* | 5'-AGGTTGTCTCCTGCGACTTCA-3' (Forward) |
|  | 5'-TGGTCCAGGGTT TCTTACTCC-3' (Reverse) |

**Table S3**. **Biochemical characteristics of control and T2D mice in the presence or absence of NKAα1**.

| Characteristics | WT | NKAα1^+/-^ | WT+HFD | NKAα1^+/-^+HFD |
| --- | --- | --- | --- | --- |
| Body weight | 28.1±1.6 | 29.2±1.2 | 39.1±1.9* | 38.5±1.5* |
| FBG (mmol/l) | 5.5±0.4 | 5.5±0.6 | 10.1±1.2* | 10.3±1.1* |
| Insulin (ng/ml) | 2.1±0.3 | 2.2±0.4 | 7.7±0.8* | 7.6±0.9* |
| Total cholesterol (mmol/l) | 2.4±0.2 | 2.2±0.3 | 3.4±0.3* | 3.5±0.4* |
| Triacylglycerols (mmol/l) | 1.2±0.1 | 1.3±0.1 | 2.8±0.2* | 2.7±0.3* |

Notes: WT, wild-type; FBG, fasting blood glucose. Mean ± SD, n = 6. **P* < 0.05 versus WT. Differences between groups were assessed with ANOVA followed by Bonferroni post-hoc test.

**Table S4**. **Biochemical characteristics of control and T2D mice after overexpression of NKAα1**.

| Characteristics | Vector | NKAα1 OE | Vector+HFD | NKAα1 OE+HFD |
| --- | --- | --- | --- | --- |
| Body weight | 29.5±1.5 | 30.3±1.4 | 39.8±1.7* | 39.1±1.5* |
| FBG (mmol/l) | 5.7±0.6 | 5.9±0.5 | 11.1±0.7* | 12.1±0.8* |
| Insulin (ng/ml) | 2.3±0.2 | 2.1±0.4 | 7.6±0.8* | 7.8±0.9* |
| Total cholesterol (mmol/l) | 2.1±0.2 | 2.1±0.3 | 3.6±0.3* | 3.5±0.4* |
| Triacylglycerols (mmol/l) | 1.3±0.2 | 1.2±0.3 | 2.9±0.3* | 2.8±0.2* |

Notes: FBG, fasting blood glucose. Mean ± SD, n = 6. **P* < 0.05 versus Vector. Differences between groups were assessed with ANOVA followed by Bonferroni post-hoc test.

**Table S5**. **Compound information used for screening experiments.**

| **No.** | **Compounds** | **Restoration rate of NKA activity (%)** |
| --- | --- | --- |
| 1 | 5,6-Dimethylbenzimidazole | 15.5±1.3 |
| 2 | 1-Naphthaleneacetic acid | 8.9±0.9 |
| 3 | 10-Hydroxydecanoic Acid | 11.7±0.8 |
| 4 | D-(+)-Galacturonic acid | 1.3±0.2 |
| 5 | Mandelic acid | 2.5±0.3 |
| 6 | Indole-3-acetic acid | 7.9±0.5 |
| 7 | Oxalic acid | 28.8±3.1 |
| 8 | 4-Methoxysalicylic acid | 52.2±4.8 |
| 9 | Bisdemethoxycurcumin | 21.3±2.6 |
| 10 | Oroxin A | 17.8±1.3 |
| 11 | Procyanidin B1 | 27.8±2.5 |
| 12 | Hispidulin | 32.8±3.1 |
| 13 | 4',6,7-Trimethoxyisoflavone | 38.8±3.3 |
| 14 | Rhamnocitrin | 45.4±4.8 |
| 15 | Hamaudol | 89.2±3.4 |
| 16 | Gossypin | 45.1±3.6 |
| 17 | Dihydrodaidzein | 22.2±2.5 |
| 18 | Aromadendrin | 67.3±4.6 |
| 19 | 6,7,4'-Trihydroxyisoflavone | 21.7±3.3 |
| 20 | 6-Methoxyluteolin | 34.7±2.5 |
| 21 | 8-Prenylnaringenin | 53.3±4.9 |
| 22 | Sakuranetin | 12.6±1.4 |
| 23 | Skullcapflavone II | 4.3±0.4 |
| 24 | 4',5,7-Trimethoxyflavone | 21.3±2.3 |
| 25 | (±)-Catechin hydrate | 15.5±1.9 |
| 26 | 4'-METHOXYFLAVONE | 32.6±3.9 |
| 27 | 3,4-Dihydroxyflavone | 4.2±1.2 |
| 28 | 5,7,3',4'-Tetramethoxyflavone | 1.9±0.3 |
| 29 | Norwogonin | 56.5±2.1 |
| 30 | Kaempferol 3-gentiobioside | 21.6±1.7 |
| 31 | Visnagin | 57.6±5.1 |
| 32 | 5-Hydroxyflavone | 31.4±2.7 |
| 33 | Aloeresin D | 23.4±2.1 |
| 34 | EGCG Octaacetate | 71.3±4.6 |
| 35 | Tilianin | 45.1±5.1 |
| 36 | 6-Demethoxytangeretin | 23. 1±3.7 |
| 37 | Quercetagetin | 45.4±3.8 |
| 38 | 4-METHOXYCHALCONE | 16.6±1.8 |
| 39 | 7-Hydroxyflavanone | 23.4±2.1 |
| 40 | 3-Methoxyflavone | 6.7±0.4 |
| 41 | 4-Hydroxyflavanone | 45.7±4.1 |
| 42 | 2-Hydroxyflavanone | 7.5±0.9 |
| 43 | 3,6-Dihydroxyflavone | 71.2±5.6 |
| 44 | 2-Hydroxychalcone | 65.7±4.2 |
| 45 | 4'-Hydroxychalcone | 41.2±4.8 |
| 46 | 7-Methoxyflavone | 23.5±2.7 |
| 47 | Reynoutrin | 17.5±1.3 |
| 48 | Theaflavin | 5.5±0.7 |
| 49 | 7-Hydroxyflavone | 10.8±1.1 |
| 50 | Procyanidin B2 | 23.6±1.9 |
| 51 | Apigenin-7-glucuronide | 12.3±1.4 |
| 52 | Sophoraflavanone G | 8.9±0.6 |
| 53 | Kurarinone | 34.5±4.9 |
| 54 | Kaempferol 3-glucorhamnoside | 12.5±1.3 |
| 55 | MOSLOFLAVONE | 45.6±4.2 |
| 56 | 5,7-DIMETHOXYFLAVONE | 56.8±5.2 |
| 57 | 5-hydroxy-7,8-dimethoxyflavone | 23.5±2.1 |
| 58 | Vitexia-glucoside | 17.8±0.9 |
| 59 | 5-DEMETHYLNOBILETIN | 78.1±0.61 |
| 60 | 2''-O-β-L-Galorientin | 4.3±0.54 |
| 61 | GALANGIN-3-METHYLETHER | 51.2±4.6 |
| 62 | 4',7-DIMETHOXY-5-HYDROXYFLAVONE | 12.8±1.1 |
| 63 | Maltol | 34.5±3.7 |
| 64 | Cyanidin Chloride | 28.9±2.1 |
| 65 | 3’- Methoxy Puerarin | 41.3±3.6 |
| 66 | Trifolirhizin | 3.3±0.1 |
| 67 | Quercetin-3-O-β-D-glucose-7-O-β-D-gentiobiosiden | 23.6±2.5 |
| 68 | HOMOPLANTAGININ | 7.9±1.4 |
| 69 | Isoliquiritin apioside | 32.5±3.2 |
| 70 | 7,2'-dihydroxy-3',4'-dimethoxyisoflavane-7-O-glucoside | 56.5±4.3 |
| 71 | Karanjin | 12.6±1.4 |
| 72 | 4-Hydroxycoumarin | 61.4±6.7 |
| 73 | Quercetagitrin | 5.4±0.5 |
| 74 | Procyanidin C1 | 2.4±0.3 |
| 75 | Hesperetin 7-O-glucoside | 34.4±3.8 |
| 76 | Iristectorigenin A | 21.2±2.3 |
| 77 | Isorhamnetin-3-O-glucoside | 17.7±1.5 |
| 78 | Quercimeritrin | 2.4±0.3 |
| 79 | Luteolin-3-O-beta-D-glucuronide | 12.5±1.3 |
| 80 | Neoeriocitrin | 34.5±3.1 |
| 81 | Taxifolin 7-O-rhamnoside | 0.7.±0.1 |
| 82 | Vicenin 3 | 15.6±1.1 |
| 83 | Vaccarin | 17.1±1.2 |
| 84 | Glycerol Tri-n-octanoate | 34.2±3.1 |
| 85 | Glycerol Trieicosanoate | 3.2±0.4 |
| 86 | Glycerol Tridecanoate | 41.2±5.1 |
| 87 | Glycerol trilinoleate | 27.8±2.2 |
| 88 | 1-Oleoyl-rac-glycerol | 23.4±2.8 |
| 89 | Cannabidiol | 45.6±4.3 |
| 90 | Hexahydrocurcumin | 3.4±0.4 |
| 91 | Dimethylcurcumin | 51.2±4.2 |
| 92 | Chebulinic acid | 23.2±1.1 |
| 93 | Chebulagic acid | 17.8±2.2 |
| 94 | 2',3'-Dihydroxy-4'-methoxyacetophenone | 17.6±1.9 |
| 95 | Homovanillyl alcohol | 27.7±2.4 |
| 96 | Homogentisic acid | 4.4±0.5 |
| 97 | Eugenin | 5.1±0.6 |
| 98 | Atranorin | 36.9±3.9 |
| 99 | Isopsoralenoside | 21.3±2.4 |
| 100 | Psoralenoside | 14.5±1.5 |
| 101 | Pyromeconic acid | 3.3±0.3 |
| 102 | Gigantol | 19.1±1.6 |
| 103 | Chicoric Acid | 27.4±2.8 |
| 104 | Nordihydroguaiaretic acid | 3.1±0.4 |
| 105 | Deoxyrhapontin | 1.6±0.2 |
| 106 | 2-5-dihydroxyacetophenone | 34.2±2.8 |
| 107 | Glucosyringic acid | 1.5±0.1 |
| 108 | Phenylacetaldehyde | 35.7±4.1 |
| 109 | 2'-Hydroxyacetophenone | 46.3±4.1 |
| 110 | BENZYLACETONE | 21.7±2.4 |
| 111 | trans-Benzylideneacetone | 16.6±1.5 |
| 112 | Atraric acid | 9.7±1.1 |
| 113 | 4-Methoxybenzoic acid | 1.2±0.2 |
| 114 | Forsythoside I | 0.9±0.2 |
| 115 | Raspberry ketone glucoside | 1.4±0.3 |
| 116 | 2-HYDROXY-3,4-DIMETHOXYBENZOIC ACID | 5.3±0.5 |
| 117 | androsin | 6.1±0.3 |
| 118 | Hydroxytyrosol Acetate | 21.5±2.5 |
| 119 | Cannabidivarin | 45.6±4.1 |
| 120 | Gallic aldehyde | 4.2±0.2 |
| 121 | Anisic aldehyde | 6.6±0.4 |
| 122 | 2-methoxycinnamaldehyde | 1.5±0.1 |
| 123 | 3,5-Dimethoxyphenol | 32.1±3.2 |
| 124 | 6-paradol | 3.1±0.4 |
| 125 | Gnetol | 56.6±4.5 |
| 126 | Ginkgolic acid C15:1 | 1.5±0.2 |
| 127 | Ginkgolic acid C13:0 | 2.3±0.2 |
| 128 | Ginkgolic acid C17:1 | 3.1±0.3 |
| 129 | Geraniin | 4.3±0.6 |
| 130 | Curculigoside | 3.1±0.4 |
| 131 | 6-Shogaol | 2.5±0.2 |
| 132 | 8-Gingerol | 4.1±0.3 |
| 133 | Paeonolide | 45.6±3.3 |
| 134 | Mulberroside A | 32.4±3.8 |
| 135 | Vanillin | 15.2±1.4 |
| 136 | Gossypol | 56.7±4.5 |
| 137 | Acetovanillone | 61.3±6.8 |
| 138 | D-DELTA-TOCOPHEROL | 34.4±4.3 |
| 139 | Orcinol gentiobioside | 12.3±1.7 |
| 140 | Apiopaeonoside | 56.1±4.3 |
| 141 | Desmethoxy yangonin | 12.6±1.1 |
| 142 | Rhaponiticin | 5.3±0.3 |
| 143 | 4-Ethylphenol | 6.6±0.5 |
| 144 | Isovanillic acid | 1.2±0.3 |
| 145 | 4'-Methoxyresveratrol | 13.7±1.1 |
| 146 | Methylnissolin-3-O-glucoside | 27.7±3.1 |
| 147 | Acetyl-trans-resveratrol | 4.1±0.5 |
| 148 | 3,4-Dimethoxybenzaldehyde | 6.5±0.8 |
| 149 | Zearalenone | 8.9±0.9 |
| 150 | 4-Hydroxymandelic acid | 67.2±5.3 |
| 151 | DL -3,4-Dihydroxymandelic acid | 51.3±5.2 |
| 152 | Agrimol B | 12.4±1.3 |
| 153 | Dryocrassin ABBA | 6.8±0.7 |
| 154 | Alnustone | 21.4±2.2 |
| 155 | DL-Normetanephrine hydrochloride | 4.1±0.2 |
| 156 | alpha-Arbutin | 1.9±0.3 |
| 157 | 3,4-Dihydroxyphenylacetic acid | 6.8±1.1 |
| 158 | 3-Hydroxyphenylacetic acid | 8.9±0.9 |
| 159 | 4-Methylcatechol | 12.6±1.7 |
| 160 | 3-Methoxytyramine hydrochloride | 29.6±2.1 |
| 161 | L-KAWAIN | 1.7±1.1 |
| 162 | Kakuol | 3.5±0.4 |
| 163 | Dendrophenol | 0.7±0.1 |
| 164 | Oxyresveratrol | 6.3±0.7 |
| 165 | yangonin | 54.3±5.1 |
| 166 | Thymol | 12.6±1.4 |
| 167 | Homovanillic acid | 7.5±0.6 |
| 168 | Punicalagin | 8.9±1.3 |
| 169 | 10-Gingerol | 34.5±3.2 |
| 170 | Erianin | 2.1±0.1 |
| 171 | 7,2'-Dihydroxy-3',4'-dimethoxyisoflavan | 5.5±1.5 |
| 172 | Pinosylvin | 6.1±0.9 |
| 173 | Dihydroresveratrol | 7.8±0.8 |
| 174 | Isorhapontigenin | 11.3±1.3 |
| 175 | Corilagin | 43.6±5.1 |
| 176 | 1,2,3,4,6-O-Pentagalloylglucose | 21.4±2.3 |
| 177 | Rhapontigenin | 17.5±1.5 |
| 178 | 2'-Hydroxy-5'-methoxyacetophenone | 53.5±4.9 |
| 179 | 2,6-Dimethoxybenzoic acid | 67.6±5.6 |
| 180 | Pinostilbene | 31.3±3.6 |
| 181 | 4-Hydroxybenzyl alcohol | 17.8±1.5 |
| 182 | Veratric acid | 7.6±0.7 |
| 183 | Olivetol | 43.4±5.1 |
| 184 | Tetrahydro Curcumin | 3.2±0.3 |
| 185 | Methyl gallate | 6.8±0.6 |
| 186 | Ethyl gallate | 31.4±3.7 |
| 187 | Methylparaben | 45.2±3.5 |
| 188 | Methyl syringate | 5.3±0.3 |
| 189 | β-thujaplicin | 4.2±0.3 |
| 190 | Sesamol | 7.9±0.7 |
| 191 | Helicid | 31.1±3.8 |
| 192 | 3,4-Dihydroxyphenylethanol | 5.2±0.5 |
| 193 | 6-Gingerol | 2.2±0.3 |
| 194 | Bakuchiol | 1.2±0.1 |
| 195 | Protocatechualdehyde | 0.7±0.1 |
| 196 | Honokiol | 1.9±0.2 |
| 197 | p-Hydroxybenzaldehyde | 9.3±0.6 |
| 198 | 5-Hydroxy-1,7-diphenyl-6-hepten-3-one | 48.9±5.5 |
| 199 | Isoeugenol | 32.1±3.2 |
| 200 | Chrysophanic Acid | 7.9±0.8 |
| 201 | Cardamonin | 6.8±0.5 |
| 202 | 4-Hydroxybenzoic acid | 5.1±0.4 |
| 203 | (-)-Epigallocatechin Gallate | 8.1±0.9 |
| 204 | Epigallocatechin | 7.6±0.5 |
| 205 | Xanthoxyline | 5.6±0.4 |
| 206 | Sodium Danshensu | 3.3±0.4 |
| 207 | 3,4,5-Trimethoxyphenol | 5.5±0.2 |
| 208 | Ethyl Vanillate | 23.2±2.6 |
| 209 | Paeonol | 1.6±0.1 |
| 210 | Pterostilbene | 3.7±0.2 |
| 211 | Phloretic acid | 4.2±0.5 |
| 212 | Tyrosol | 5.3±0.6 |
| 213 | Gentisic acid | 12.5±1.4 |
| 214 | Phloracetophenone | 5.1±0.3 |
| 215 | Orsellinic acid | 14.4±1.1 |
| 216 | Morin | 41.2±4.6 |
| 217 | Ethyl ferulate | 21.7±2.3 |
| 218 | Caffeic Acid | 5.3±0.5 |
| 219 | 7-Methoxy-4-methylcoumarin | 5.1±0/6 |
| 220 | Orsellinic acid ethyl ester | 7.3±0.8 |
| 221 | (+)-Catechin Hydrate | 5.1±0.2 |
| 222 | Orcinol glucoside | 4.1±0.3 |
| 223 | Rosmarinic acid | 7.1±0.6 |
| 224 | Gossypol acetic acid | 3.2±0.3 |
| 225 | Salvianolic acid B | 2.3±0.3 |
| 226 | Methylarbutin | 1.7±0.1 |
| 227 | Hematoxylin | 31.9±2.6 |
| 228 | Vitamin E | 3.1±0.3 |
| 229 | Resveratrol | 2.7±0.2 |
| 230 | Methyl protocatechuate | 23.8±2.5 |
| 231 | Terphenyllin | 21.2±3.1 |
| 232 | Guaiacol | 8.3±0.9 |
| 233 | Eugenol | 4.2±0.5 |
| 234 | Vitamin E Acetate | 23.4±2.3 |
| 235 | 3-Hydroxy-4-methoxyacetophenone | 17.8±1.2 |
| 236 | Gallic acid | 3.6±0.4 |
| 237 | Ethylparaben | 1.4±0.1 |
| 238 | Benzoic acid | 2.1±0.3 |
| 239 | Cianidanol | 4.3±0.1 |
| 240 | Tannic acid | 32.4±3.1 |
| 241 | 3,4-Dimethoxybenzyl alcohol | 18.9±1.2 |
| 242 | Gallic acid trimethyl ether | 27.8±4.6 |
| 243 | Methyl EudesMate | 3.1±0.2 |
| 244 | Protocatechuic acid | 4.3±0.3 |
| 245 | Ellagic acid | 23.1±2.7 |
| 246 | Phenylephrine hydrochloride | 14.2±1.8 |
| 247 | Methyl salicylate | 8.2±0.8 |
| 248 | Salicylamide | 5.6±0.4 |
| 249 | Methylsyringol | 21.2±1.7 |
| 250 | 4-Hydroxyphenylacetonitrile | 41.3±5.2 |
| 251 | 2'-Hydroxy-4'-methylacetophenone | 56.5±6.1 |
| 252 | Ethyl 4-hydroxyphenylacetate | 37.8±3.3 |
| 253 | 4-(4-Methoxyphenyl)-2-butanone | 8.5±0.9 |
| 254 | Methyl 4-hydroxycinnamate | 7.1±0.5 |
| 255 | Rubrofusarin-6-O-beta-D-gentiobioside | 5.7±0.3 |
| 256 | Ethyl salicylate | 34.5±3.9 |
| 257 | kaempferide | 16.8±1.2 |
| 258 | Cimifugin beta-D-glucopyranoside | 34.4±4.5 |
| 259 | 5-O-Methylvisammioside | 23.4±2.1 |
| 260 | Protohypericin | 2.5±0.2 |
| 261 | alpha-Tocopherolquinone | 3.6±0.3 |
| 262 | Embelin | 5.1±0.3 |
| 263 | Acetoxyisovalerylalkannin | 7.9±0.8 |
| 264 | Acetyl shikonin | 11.6±1.1 |
| 265 | β,β-Dimethylacrylalkannin | 21.3±2.4 |
| 266 | Alkannin | 41.3±5.2 |
| 267 | beta, beta-dimethylacrylshikonin | 8.1±0.8 |
| 268 | Lapachol | 12.5±1.2 |
| 269 | Juglone | 8.9±0.8 |
| 270 | Dimethylacrylshikonin | 63.3±6.7 |
| 271 | 2-Methoxynaphthoquinone | 31.2±3.6 |
| 272 | Rheic Acid | 45.3±3.4 |
| 273 | Lawsone | 16.8±1.4 |
| 274 | Alizarin | 5.2±0.6 |
| 275 | Aloe-emodin | 32.6±3.5 |
| 276 | Plumbagin | 1.4±0.1 |
| 277 | Ketoisophorone | 19.7±1.2 |
| 278 | Vitamin K1 | 16.6±1.5 |
| 279 | Antrapurol | 23.5±2.9 |
| 280 | Menadione | 19.6±1.2 |
| 281 | Desacetylcinobufagin | 1.8±0.2 |
| 282 | Sitostenone | 23.1±2.4 |
| 283 | Taurodeoxycholate sodium salt | 45.5±5.1 |
| 284 | Sodium taurochenodeoxycholate | 24.6±2.1 |
| 285 | Fucosterol | 5.1±0.3 |
| 286 | Pennogenin 3-O-beta-chacotrioside | 4.5±0.2 |
| 287 | Qingyangshengenin | 23.4±2.7 |
| 288 | Polyphyllin VI | 15.6±1.8 |
| 289 | Tenacissoside H | 31.6±3.6 |
| 290 | Tenacissoside I | 3.4±0.3 |
| 291 | Tenacissoside G | 9.4±0.8 |
| 292 | Ruscogenin | 51.3±5.2 |
| 293 | Ophiopogonin D | 15.6±1.1 |
| 294 | Dioscin | 45.5±4.2 |
| 295 | Periplocin | 34.5±3.8 |
| 296 | Prosapogenin A | 12.6±1.5 |
| 297 | Pseudoprotodioscin | 6.9±0.8 |
| 298 | Bufotaline | 5.1±0.7 |
| 299 | Arenobufagin | 43.6±0.5 |
| 300 | Tigogenin | 16.6±1.7 |
| 301 | (25RS)-Ruscogenin | 9.6±0.8 |
| 302 | Guggulsterone E&Z | 1.1±0.7 |
| 303 | GLYCODEOXYCHOLIC ACID | 6.6±0.5 |
| 304 | Periplogenin | 4.2±0.5 |
| 305 | Hecogenin | 2.5±0.2 |
| 306 | TOMATIDINE HYDROCHLORIDE | 1.7±0.3 |
| 307 | Officinalisinin I | 34.5±3.8 |
| 308 | Liriopesides B | 14.5±1.5 |
| 309 | Polyphyllin I | 12.2±1.1 |
| 310 | Diosgenin glucoside | 43.4±0.5 |
| 311 | Sitogluside | 4.4±0.2 |
| 312 | Cyasterone | 3.6±0.3 |
| 313 | Liriope muscari baily saponins C | 2.7±0.2 |
| 314 | Gracillin | 45.4±4.1 |
| 315 | Methyl protodioscin | 21.5±2.6 |
| 316 | Cinobufagin | 1.6±0.3 |
| 317 | Timosaponin AIII | 3.6±0.2 |
| 318 | Timosaponin BII | 3.9±0.3 |
| 319 | Euphorbiasteroid | 4.6±0.6 |
| 320 | Brassinolide | 34.3±3.9 |
| 321 | Caudatin | 12.5±1.8 |
| 322 | Hyodeoxycholic acid | 45.6±4.9 |
| 323 | Beta-Sitosterol | 22.5±2.5 |
| 324 | Deoxycholic acid | 18.6±1.9 |
| 325 | Cholic Acid | 4.4±0.6 |
| 326 | Cholesteryl Acetate | 2.2±0.2 |
| 327 | 5alpha-Cholestan-3-one | 6.5±0.7 |
| 328 | Cortisone | 12.3±1.2 |
| 329 | Hydroxyecdysone | 32.3±3.4 |
| 330 | Glycocholic acid | 1.5±0.2 |
| 331 | Asiatic acid | 4.9±0.3 |
| 332 | Madecassic acid | 23.7±2.1 |
| 333 | Cortodoxone | 5.2±0.6 |
| 334 | Protodioscin | 5.1±0.4 |
| 335 | Adrenosterone | 2.2±0.1 |
| 336 | Lithocholic acid | 34.8±3.5 |
| 337 | Epiandrosterone | 11.9±1.3 |
| 338 | Bufalin | 70.7±7.8 |
| 339 | 5Beta-Pregnane-3Alpha,20alpha-Diol | 54.4±5.1 |
| 340 | Hydrocortisone | 34.±3.1 |
| 341 | Estriol | 5.2±0.5 |
| 342 | Estradiol | 4.1±0.2 |
| 343 | Aquacrine | 56.5±0.4 |
| 344 | Dehydroepiandrosterone | 2.4±0.2 |
| 345 | Pregnenolone | 16.5±1.1 |
| 346 | Chenodeoxycholic acid | 27.3±2.4 |
| 347 | Lactulose | 12.4±1.4 |
| 348 | Progesterone | 42.2±4.7 |
| 349 | 7-Ketocholesterol | 2.4±0.3 |

Notes: Mean ± SD, n = 6.

**Table S6**. **Biochemical characteristics of control and T2D mice after Hamaudol treatment**.

| Characteristics | Con | Hamaudol | HFD | Hamaudol+HFD |
| --- | --- | --- | --- | --- |
| Body weight | 28.9±1.4 | 30.2±1.4 | 40.1±1.9* | 39.2±1.8* |
| FBG (mmol/l) | 5.4±0.6 | 5.6±0.4 | 11.2±0.5* | 10.8±0.5* |
| Insulin (ng/ml) | 2.3±0.2 | 2.5±0.4 | 7.9±0.4* | 8.1±0.8* |
| Total cholesterol (mmol/l) | 2.5±0.4 | 2.4±0.5 | 3.6±0.3* | 3.8±0.4* |
| Triacylglycerols (mmol/l) | 1.1±0.2 | 1.2±0.1 | 2.9±0.3* | 2.8±0.2* |

Notes: FBG, fasting blood glucose. Mean ± SD, n = 6. **P* < 0.05 versus Con. Differences between groups were assessed with ANOVA followed by Bonferroni post-hoc test.
